# AlphaFold Ensemble Competition Screens Enable Peptide Binder Design with Single-Residue Sensitivity

**DOI:** 10.1021/acschembio.4c00418

**Published:** 2024-09-18

**Authors:** Pernille Vosbein, Paula Paredes Vergara, Danny T. Huang, Andrew R. Thomson

**Affiliations:** † School of Chemistry, University of Glasgow, Glasgow G12 8QQ, U.K.; ‡ Cancer Research UK Scotland Institute, Garscube Estate, Switchback Road, Glasgow G61 1BD, U.K.; § School of Cancer Sciences, University of Glasgow, Glasgow G61 1QH, U.K.

## Abstract

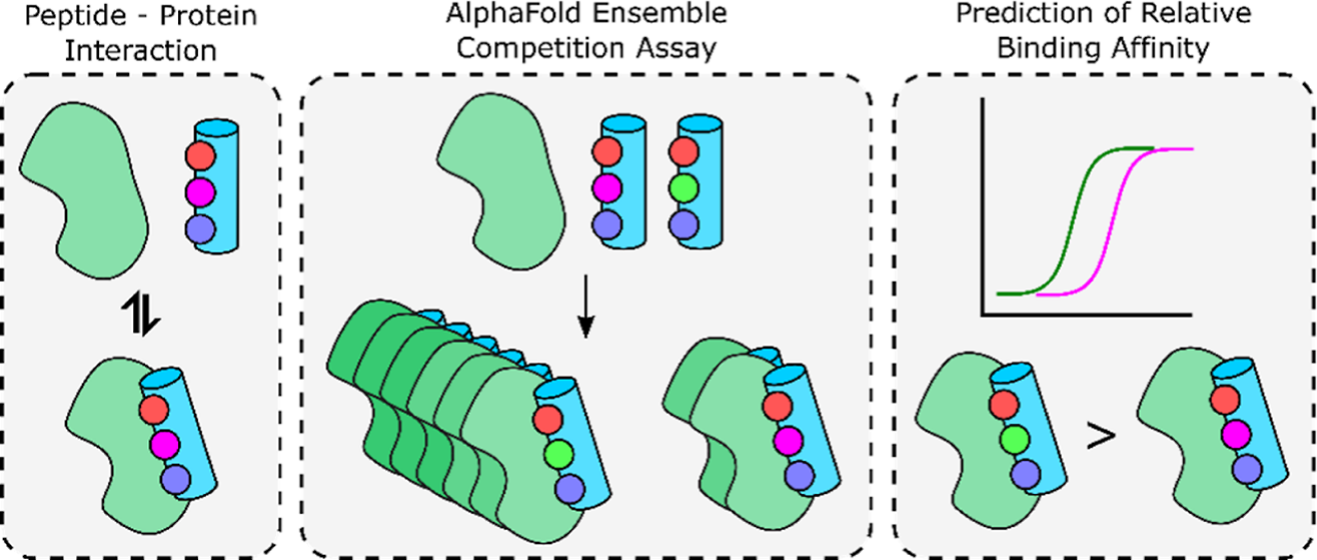

Understanding the relationship between the sequence and binding energy in peptide–protein interactions is an important challenge in chemical biology. A prominent example is ubiquitin interacting motifs (UIMs), which are short peptide sequences that recognize ubiquitin and which bind individual ubiquitin proteins with a weak affinity. Though the sequence characteristics of UIMs are well understood, the relationship between the sequence and ubiquitin binding affinity has not yet been fully characterized. Herein, we study the first UIM of Vps27 as a model system. Using an experimental alanine scan, we were able to rank the relative contribution of each hydrophobic residue of this UIM to ubiquitin binding. These results were correlated with AlphaFold displacement studies, in which AlphaFold is used to predict the stronger binder by presenting a target protein with two potential peptide ligands. We demonstrate that by generating large numbers of models and using the consensus bound-state AlphaFold competition experiments can be sensitive to single-residue variations. We furthermore show that to fully recapitulate the binding trends observed for ubiquitin, it is necessary to screen AlphaFold models that incorporate a “decoy” binding site to prevent the displaced peptide from interfering with the actual binding site. Overall, it is shown that AlphaFold can be used as a powerful tool for peptide binder design and that when large ensembles of models are used, AlphaFold predictions can be sensitive to very small energetic changes arising from single-residue alterations to a binder.

## Introduction

Peptide–protein interactions are vital in regulating many biological processes, but the relationship between the peptide sequence and binding affinity can be challenging to predict a priori.^1,2^ Interactions between proteins and α-helical peptides have been extensively studied in this context as potentially tractable systems for applications in chemical biology.^3,4^ Contemporary machine learning methods for the protein complex structure prediction–most notably AlphaFold^5^ and AlphaFold Multimer^6^—are now able to reliably predict the geometry of peptide–protein complexes and in some cases to predict the stronger binder from a binary choice of two competing peptide ligands.^7^ Discrimination between single residue alterations in a peptide ligand sequence has, to our knowledge, not yet been demonstrated. These methods could function as a means of engineering or even designing de novo binders for target proteins. This has the potential to be a transformative technology in chemical biology, where control of the binding affinity of a peptide ligand can enable precise dissection of the energetics of binding events. Herein, we explore the application of this technique to the well-studied ubiquitin-UIM binding event.

Ubiquitin (Ub) is a 76 residue protein that is post translationally added to other proteins as a means of marking them for processing within the cell.^8−10^ Ub recognition is complex, and several small protein and peptide motifs are known to bind Ub.^11^ Ub binding is generally characterized by the formation of multiple weak interactions with individual Ub folds within a more-complex binding event in which polyUb chains are recognized.^12^ Understanding the recognition of polyUb chains would, therefore, benefit from a better understanding of the energetics of individual Ub binding motifs. In a more general sense, an appreciation of the energetic consequences of single-residue modifications to a protein binding peptide motif will enable the design of peptidic systems for chemical biology and beyond.

Ubiquitin interacting motifs (UIMs) are one of the most extensively characterized of the small Ub binding domains.^13−15^ These sequences recognize Ub through a single α-helix that binds in the hydrophobic patch of the β-sheet region of Ub. UIMs are characterized by a central “AxxxS” motif that is flanked by hydrophobic and negatively charged residues. Ub binding affinities for isolated UIMs have been reported in the low millimolar to high micromolar range. We chose one of the more-studied examples of this motif, the first UIM of the Vps27 protein, hereafter referred to as UIM-1, as an experimental system.^16^ Herein, we use this peptide:protein interaction as a model system to investigate the capacity of AlphaFold Multimer to predict the effect on Ub affinity of changes to the UIM-1 peptide sequence.

## Results and Discussion

### Preliminary Peptides

**Figure 1:**
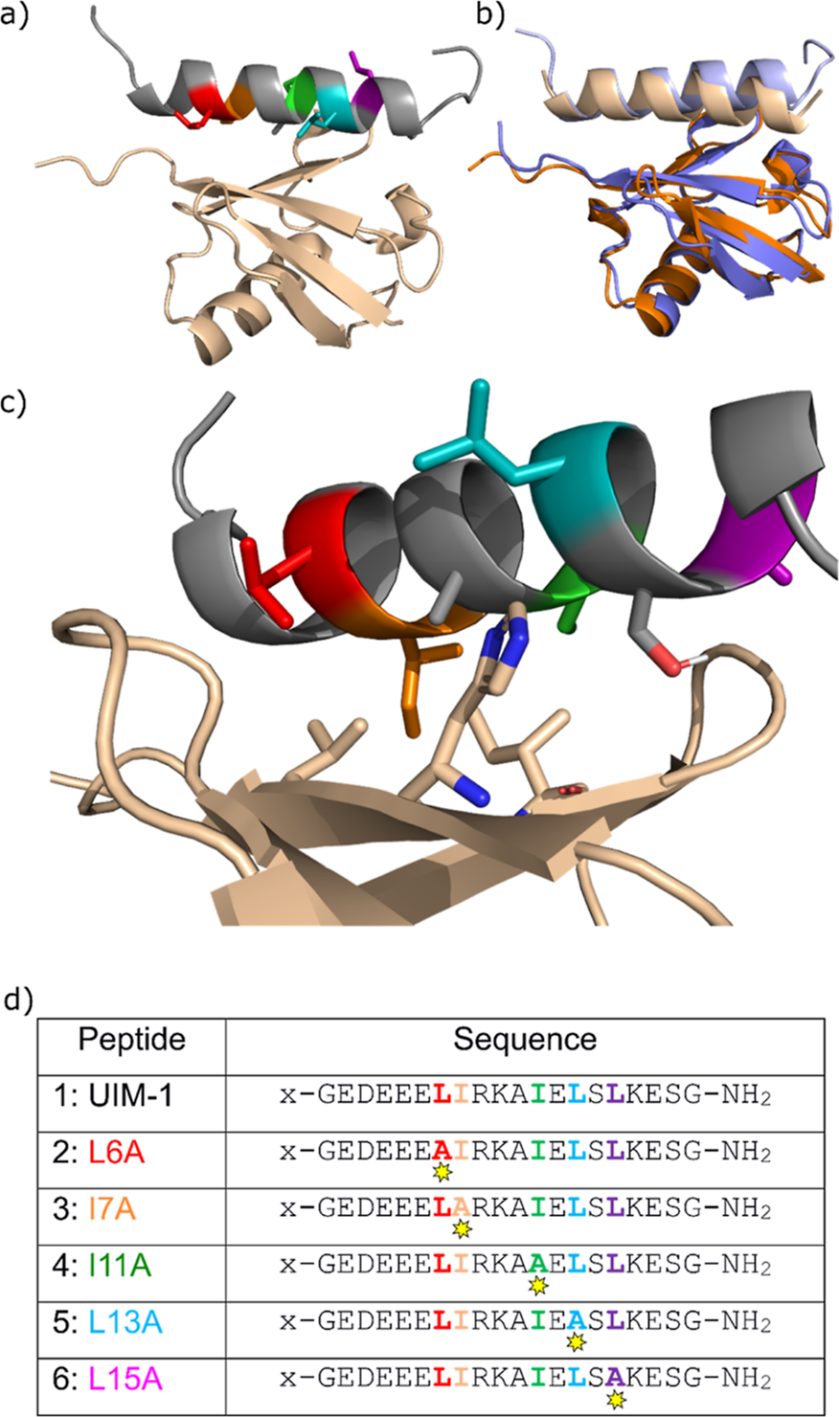
a) NMR structure (pdb: 1q0w) of Ub:Vps27 UIM-1 complex. Ub is shown in beige and UIM-1 in gray, with hydrophobic residues highlighted in rainbow colors. (b) NMR structure (blue and pale blue) overlaid with an AlphaFold model of Ub:UIM-1 complex (orange and pale yellow). (c) Detail of the Ub:UIM-1 interaction, showing key amino acid side chains. (d) Sequences for UIM-1 and alanine mutants. All peptides were prepared as labeled (x = FITC-β-alanyl) and capped (x = acetyl-β-alanyl) versions, denoted **a** and **b** variants, respectively. For full details, see Supporting Information, Tables S1 and S2.

We selected the UIM-1 sequence as an experimental model system because it is well characterized, having both the reported dissociation constant and NMR structure (pdb: 1q0w, Figure 1a,c). A dissociation constant of 277 μmol·L^–1^ has been reported for this complex.^16^ The UIM-1 sequence binds to Ub primarily through a series of hydrophobic contacts around the characteristic “AxxxS” UIM pattern of alanine and serine residues spaced four positions apart. Further, polar and salt bridge contacts may also stabilize the complex. UIM-1 notably also has a series of i–i+4 salt bridges across the non Ub binding “back” face of the helix, which likely contribute indirectly to Ub binding by stabilizing the helical form of the peptide.

We chose to investigate peptides based on residues 257–274 of the UIM-1 sequence, which represents the core helical region of contact between the UIM and Ub (Figure 1a,d). In both our computational and experimental studies, this core sequence was flanked with capping glycine residues in order to minimize end effects. Synthetic peptides were, furthermore, N-terminally acetyl capped or fluorescein labeled and C-terminally amidated. AlphaFold Multimer does not currently represent acetyl caps or C-terminal amidations, and these features were omitted from the AlphaFold screens (see below). Initially, we checked whether AlphaFold Multimer could replicate the bound state of this peptide. The RMSD for the overlay of the AlphaFold model of the Ub:UIM-1 complex was 0.83 Å, demonstrating that AlphaFold can reliably model this complex (Figure 1b). Taking this into account we designed, sequences **1**–**6**, in which the core sequence of UIM-1 is either preserved or in which the hydrophobic residues are systematically changed to alanine (Figure 1d).

### Anomalously Strong Binding of Fluorescein-Labeled Peptides

**Figure 2:**
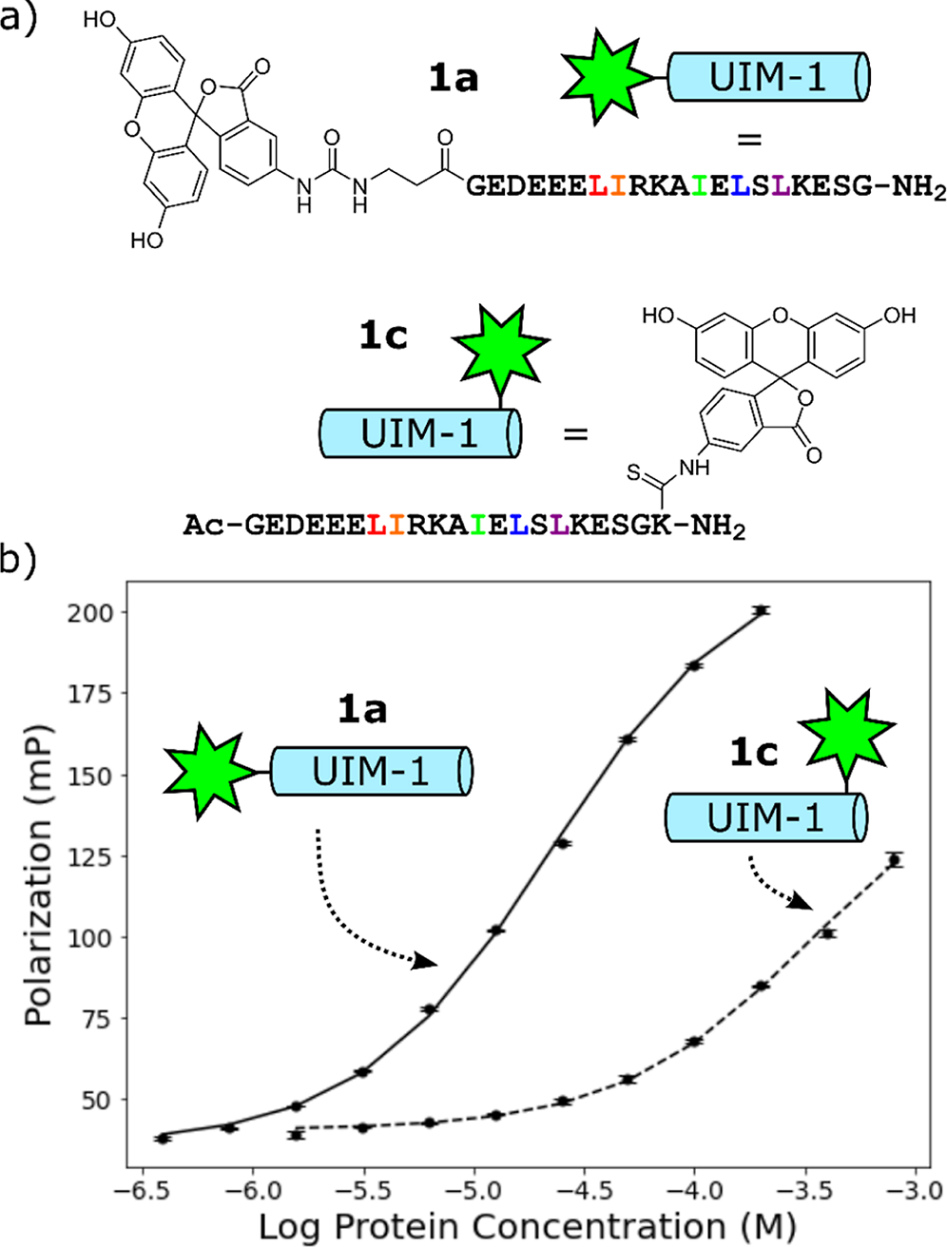
a) Schematic and cartoon representations of label positioning in peptides **1a** and **1c**. (b) FP titration traces for **1a** and **1c** against Ub, showing the effect of label position on the binding affinity.

We initially constructed peptide **1a**, in which a fluorescein label is appended to the N-terminus of the core UIM-1 sequence via a β-alanine spacer. The spacer acts to distance the fluorophore from the binding motif and, furthermore, prevents Edman degradation during the TFA cleavage step of the solid-phase synthesis of **1a**.^17^ We additionally synthesized peptide **1b**, in which the fluorophore was replaced with a simple acetyl cap for circular dichroism (CD) spectroscopy measurements. Peptide **1a** was titrated against Ub in a fluorescence polarization (FP) assay. The peptide concentration was maintained at a constant of 50 nmol·L^–1^, and Ub concentration was varied by serial dilution from 200 μmol·L^–1^ to 390 nmol·L^–1^. A clear sigmoidal change in the FP signal was observed, and least-squares fitting of these data to a 1:1 binding model revealed a *K*_d_ of 21.5 ± 1.4 μmol·L^–1^ (Figure 2). This *K*_d_ is an order of magnitude lower than a literature value of 277 μmol·L^–1^, which is obtained from an NMR titration experiment of a slightly longer variant of the same peptide sequence. We hypothesized that this anomalously strong binding was due to additional interactions of the fluorescein label of peptide **1a** with the flexible *C*-terminal -RLRGG tail of Ub. To test this, we synthesized peptide **1c**, in which the fluorescein label was relocated to the side chain of a *C*-terminal lysine residue. Titration of this peptide against Ub revealed a much weaker binding with a *K*_d_ of 311.3 ± 15.5 μmol·L^–1^, corresponding to a 6.5 kJ mol^–1^ reduction in binding energy (Figure 2). Peptides **1a** and **1c** exhibit the same degree of α-helical content as determined by CD spectroscopy, indicating that the position of the fluorophore does not significantly influence the peptide secondary structure (Supporting Information Figure S1). The differences in Ub affinity are therefore most probably due to Ub-fluorophore interactions that are present with peptide **1a** but not with peptide **1c**.

These data provide a quantitative demonstration that labels such as fluorophores can significantly alter the binding behavior of the system on which they report. This finding also indicates that interactions involving the *C*-terminal tail region of Ub can contribute significantly to the energy of Ub binding events, which may have relevance to Ub binding studies in a more general sense. The purpose of our subsequent experiments was to investigate the prediction and measurement of free energy changes related to the core UIM-1 sequence. The fluorescein label is ca. 20 Å away from the core of the UIM-1 sequence, suggesting that it is likely to have a systematic influence on all the peptides in this study. The *N*-terminally fluorescein-labeled peptides are synthetically more accessible than their side chain-labeled analogues, and their tighter Ub binding enables Ub titrations to be carried out using significantly lower Ub concentrations than would otherwise be possible. With these factors in mind, we accepted the stronger Ub binding exhibited by the *N*-terminally-labeled peptide as a feature rather than a bug and continued to use this labeling position to investigate systematic substitution of alanine for hydrophobic residues within the UIM-1 sequence.

### Experimental Alanine Scan

**Figure 3:**
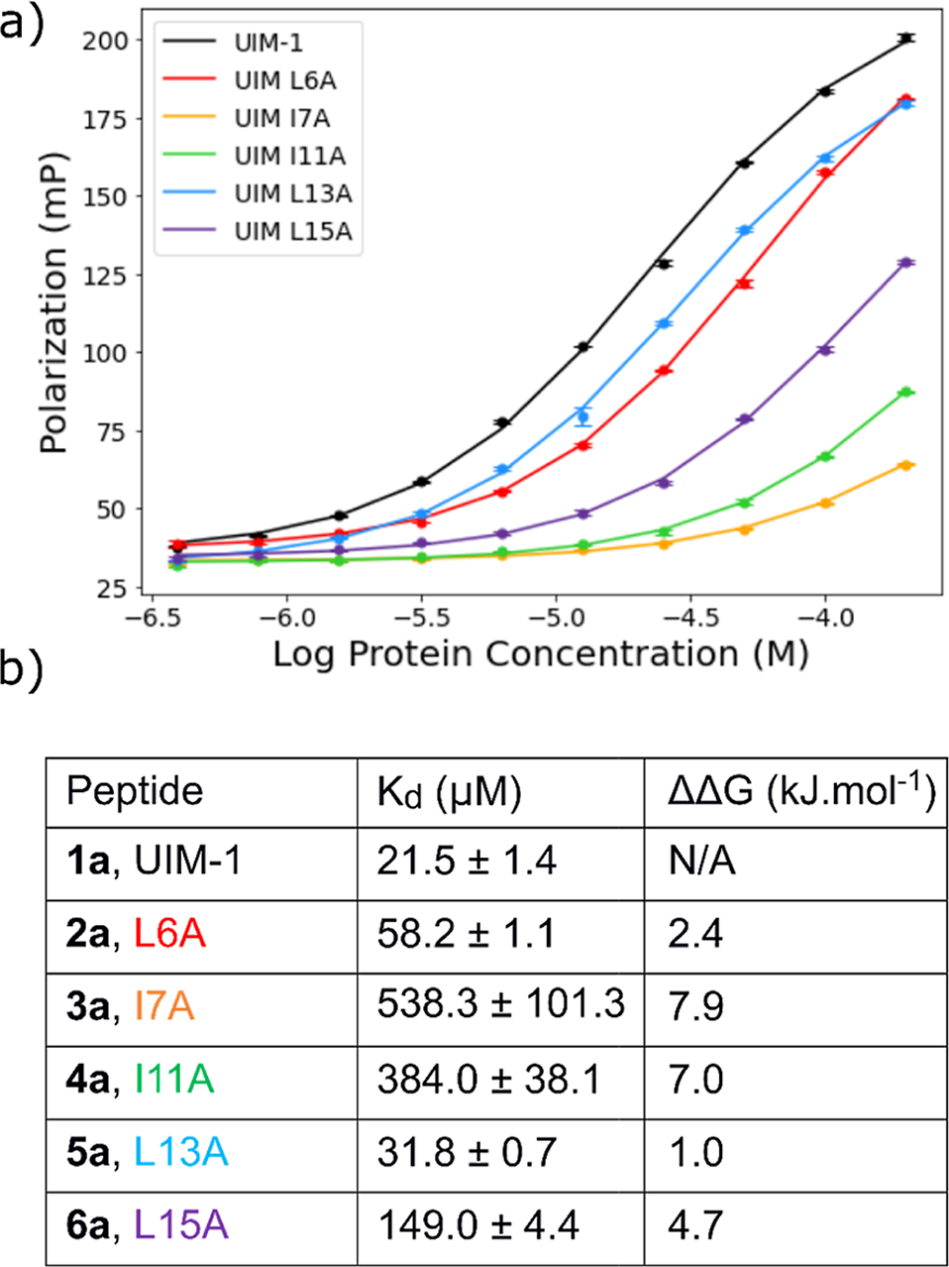
(a) FP titration curves for peptides **1a–6a** against Ub. (b) *K*_d_ and ΔΔ*G* values for binding Ub. All experiments were for *N*-terminally labeled peptides. Titrations were conducted in triplicate.

An experimental alanine scan was conducted, for which we synthesized peptides **2**–**6** (Figure 1d). As before each sequence was synthesized in a fluorescein-labeled “**a**” version for FP titrations, and an acetyl-capped “**b**” version for CD spectroscopy studies. FP titrations of fixed 50 nmol·L^–1^ concentrations of the peptide against varying concentrations of Ub were used to determine the *K*_d_ for Ub binding (Figure 3). Clear differences were seen in the Ub binding properties of peptides **2a**–**6a**. The two most deleterious mutations in terms of binding were the I7A and I11A modifications (**3a** and **4a**, respectively). Full sigmoidal binding curves were not observed for these mutants within the experimental Ub concentration range. For these curves, *K*_d_ values were obtained by assuming that the change in polarization on Ub binding will be the same as for peptide **1a**. The weaker Ub binding of peptides **3a** and **4a** is consistent with the published structure, for which the Ile7 and Ile11 residues directly face the Ub and are, therefore, most buried on complex formation. The measured contribution per residue to Ub binding, therefore, follows the order Ile7 > Ile11 > Leu15 > Leu6 > Leu13.

CD spectroscopy of the individual peptides showed each peptide to be partially helical, with some slight variations (Supporting Information, Figure S2). Specifically, the L6A variant showed almost identical helicity to the UIM-1 sequence, whereas the other mutants were ∼80% as folded. The slightly lower helicity of the variants other than L6A might reflect the loss of stabilizing i–i+4 hydrophobic interactions. The reduced preorganization toward the helical state is consistent with weaker binding to Ub, but no clear relationship was observed between helicity and *K*_d_.

### AlphaFold Competition Alanine Scan

The recent literature describes the use of AlphaFold Multimer competition studies to predict the stronger binding peptide in a binary choice between competing peptide binders of proteins.^18^ Despite not directly predicting the free energy associated with a peptide–protein complex, AlphaFold Multimer’s predictions have been shown the majority of the time to place the stronger binder in the binding site, with the weaker binding peptide located elsewhere on the surface of the target protein. Initial reports on this method conclude that AlphaFold does not perform reliably for single residue mutations but that for peptide–protein complexes with a significant difference in binding energy AlphaFold is useful for predicting the stronger binder. It was also reported that predictions were more reliable for complexes in which the peptide binder adopts a well-defined secondary structure. With these factors in mind, we explored the use of AlphaFold Multimer competition experiments to predict relative affinity in Ub:UIM complexes.

**Figure 4:**
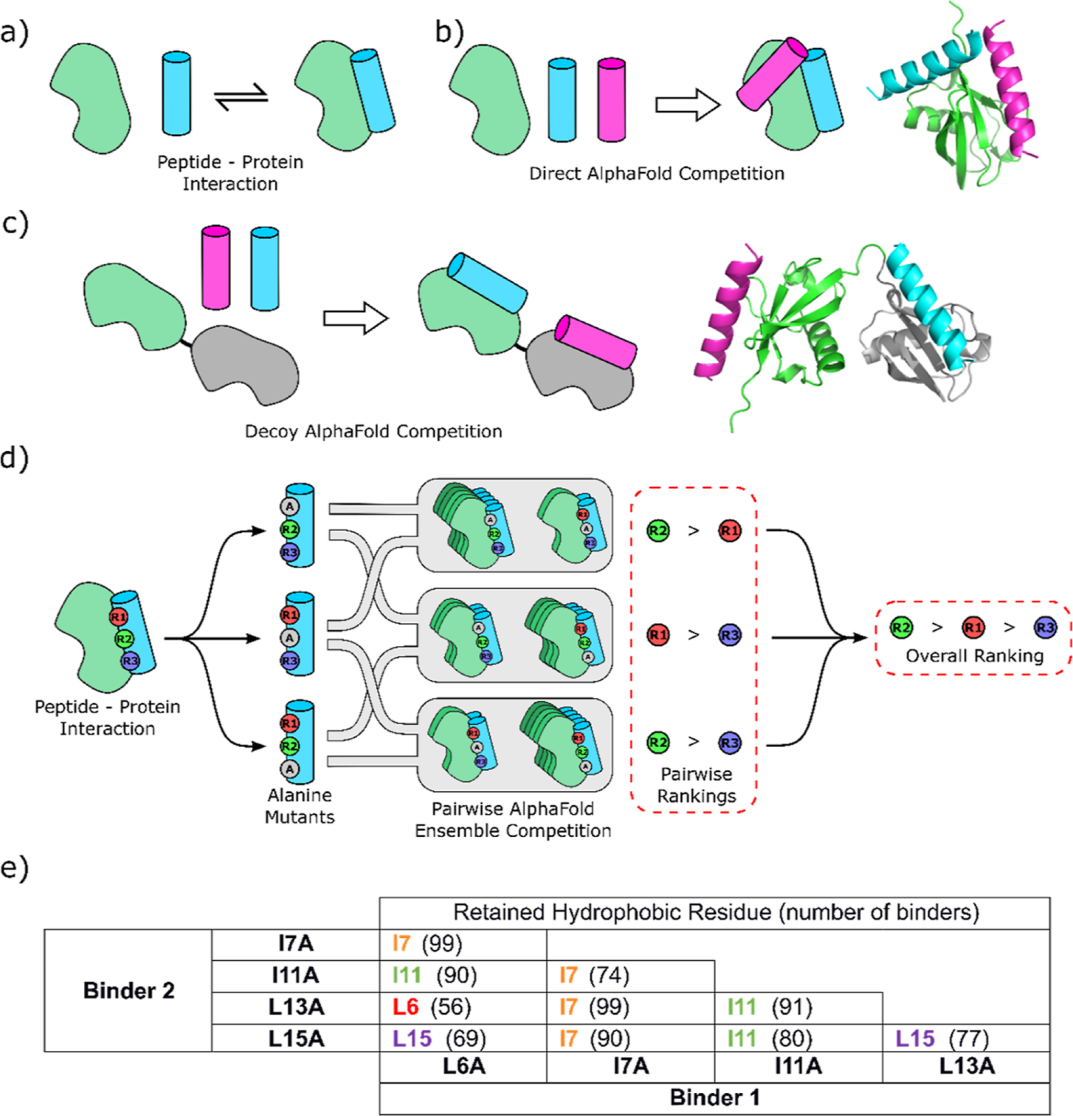
(a) Schematic of the Ub:UIM-1 interaction. (b) Schematic and representative output structure for the direct AlphaFold competition assay. (c) Schematic and representative structure for “decoy” AlphaFold competition assay, with the compromised “decoy” binding site shown in gray, with the displaced weaker binding peptide in cyan. (d) Schematic of residue ranking via pairwise AlphaFold competition assays. (e) AlphaFold competition ranking results for the UIM-1 alanine scan. Identified binders are shown with rainbow color coding as in Figure 1. Aggregate number of binding helices out of 100 possible total are given in parentheses and represent sum of states for top 50 binders each for the two Ub/decoy configurations.

We tested AlphaFold competition experiments to see whether they could replicate the experimental ranking of our alanine mutants (Figure 4). Our strategy here and for the experiments described below was to run a competition screen for every pairwise combination of alanine mutants for binding to Ub. This strategy allowed us to benchmark the AlphaFold screens against our experimental alanine scan, ahead of investigating other potential nonalanine mutants. In any pairwise comparison between alanine mutants, the mutant that retains the residue that makes the most significant contribution to the binding energy should be observed to bind the target protein. Collating the outcome of all possible pairwise competition events gives a complete ranking of the contribution to binding energy for all residues investigated. This is shown illustratively in Figure 4d, where the separate pairwise rankings of R2 > R1, R1 > R3, and R2 > R3 give the overall ranking R2 > R1 > R3. In our actual ranking experiments, the overall ordering can be determined simply by counting the number of occurrences of the retained hydrophobic residue in the tabulated results (Figure 4e).

Consistent with the reports on this method, our initial displacement experiments (for which five models were generated using the default parameters for AlphaFold Multimer as implemented in ColabFold Local) did not correlate well with our experimental data.^19^ We reasoned that a weak preference for two very similar ligands might be masked by experimental noise. We, therefore, hypothesized that greater sensitivity could be afforded by collating the results of a large number of individual competition experiments. Because of the small size of Ub, we found that generating 100 models of each complex was trivial for a GPU-accelerated workstation (ca. 5 s per model, ca. 75 min for all pairwise combinations in a full comparison run for five mutation positions). Good quality metrics were observed for all models generated, with the lowest pLDDT values typically being >70. Out of caution, we analyzed only the top 50 out of 100 models according to the AlphaFold Multimer metric. Models were analyzed with a custom Python script to identify which UIM helix was bound to Ub (see the Supporting Information). This protocol improved the correlation with our experimental data, but the rank ordering of mutants was not completely recapitulated: the L15A mutant was ranked as the least significant change, whereas our experimental studies show this to be the third most important binding residue (Supporting Information, Table S3).

### AlphaFold Competition Alanine Scan: MDM2/X Binders

To further explore this protocol, we additionally tested it against a comparable binding event for which there is published experimental alanine scan data. Binding of the MDM2 or MDMX proteins by the α-helical pMI peptide has been extensively characterized.^20^ We, therefore, ran an analogous ranking screen for the alanine mutants of the four hydrophobic binding residues of this peptide (Supporting Information, Tables S4 and S5). This study fully replicated the observed binding order, including the difference in energetic ranking of the pMI binding residues between MDM2 and MDMX. In contrast to the Ub-UIM models, a much lower variation was observed for which peptide bound, with all 50 top ranked models being consistent in all but one comparison. The pMI/MDM2/X interaction has measured *K*_d_ values in the nmol·L^–1^ range and is therefore much stronger than the interaction between UIMs and Ub, for which *K*_d_ values are typically in the high micromolar range. This suggests that AlphaFold Multimer can be sensitive to single residue alterations and that this may be less challenging to detect, in cases where there is a large overall binding energy.

### AlphaFold Competition Alanine Scan: Decoy Ub

We noted that in our AlphaFold models, the displaced peptide tended to be associated with the alternative hydrophobic patch involving Ile36 and Leu71 of Ub. Because of the small size of Ub, contacts between bound peptides were formed in the majority of the competition models (Figure 4b). We hypothesized that these contacts could be a confounding factor and, therefore, tested an alternative protocol in which a “decoy” binding site was included to prevent ligand–ligand interactions. In experimental studies, the Ile44 to Ala mutation is known to compromise the ability of Ub to bind to known Ub recognition domains.^21^ Preliminary AlphaFold studies using an I44A decoy domain did not correlate well with our experimental results (Supporting Information, Table S6). We reasoned that this was because the I44A mutation preserves the overall hydrophobic character of the binding site and, therefore, tested the I44S mutation as a hydrophobic to hydrophilic switch of the central Ile44 residue of Ub. To this end, we modeled the complex between pairs of peptide binders with a linear di-Ub chain, in which the binding groove of one “decoy” Ub domain was compromised by mutating the key Ile44 residue to serine (Figure 4c). In order to avoid biases deriving from the fusion of the binding and decoy Ub folds, both permutations were modeled (decoy *N*-terminal and decoy *C*-terminal). As before, we generated 100 models of the Ub/UIM competition complexes for this system. As intended, this arrangement leaves the bound peptides spatially separated (Figure 4c). The two Ub domains are in contact with each other in a consistent manner. In a small number of cases (a maximum of 3 in 100 models), the displaced helix was not found to associate with the decoy I44S Ub binding site, and such models were removed from the analysis. Using this protocol, we found that the stronger binding peptide was present in the unmodified binding site the majority of the time. Slight differences were noted between the ratio of bound states for the Ub-decoy and decoy-Ub screens, but no systematic variation was observed (Supporting Information, Tables S7 and S8). Overall, the combined ranking scan comprising the top 50 models for both Ub/decoy permutations completely recapitulated our experimental ranking of Ile7 > Ile11 > Leu15 > Leu6 > Leu13 (Figure 4e and Supporting Information, Table S9). We additionally tested the decoy approach in the MDM2/X system (Supporting Information, Tables S10 and S11) and found that, in this case, the sensitivity of the screen was slightly reduced, with a single competing pair misassigned. These results suggest that the inclusion of a decoy domain should be used cautiously and most likely only in situations where the two potential peptide ligands contact the target protein as well as each other. Overall, these results indicate that the AlphaFold Multimer has the capacity to be sensitive to point mutations, even where energetic differences are less than 1 kJ mol^–1^.

### AlphaFold Models for Design

**Figure 5:**
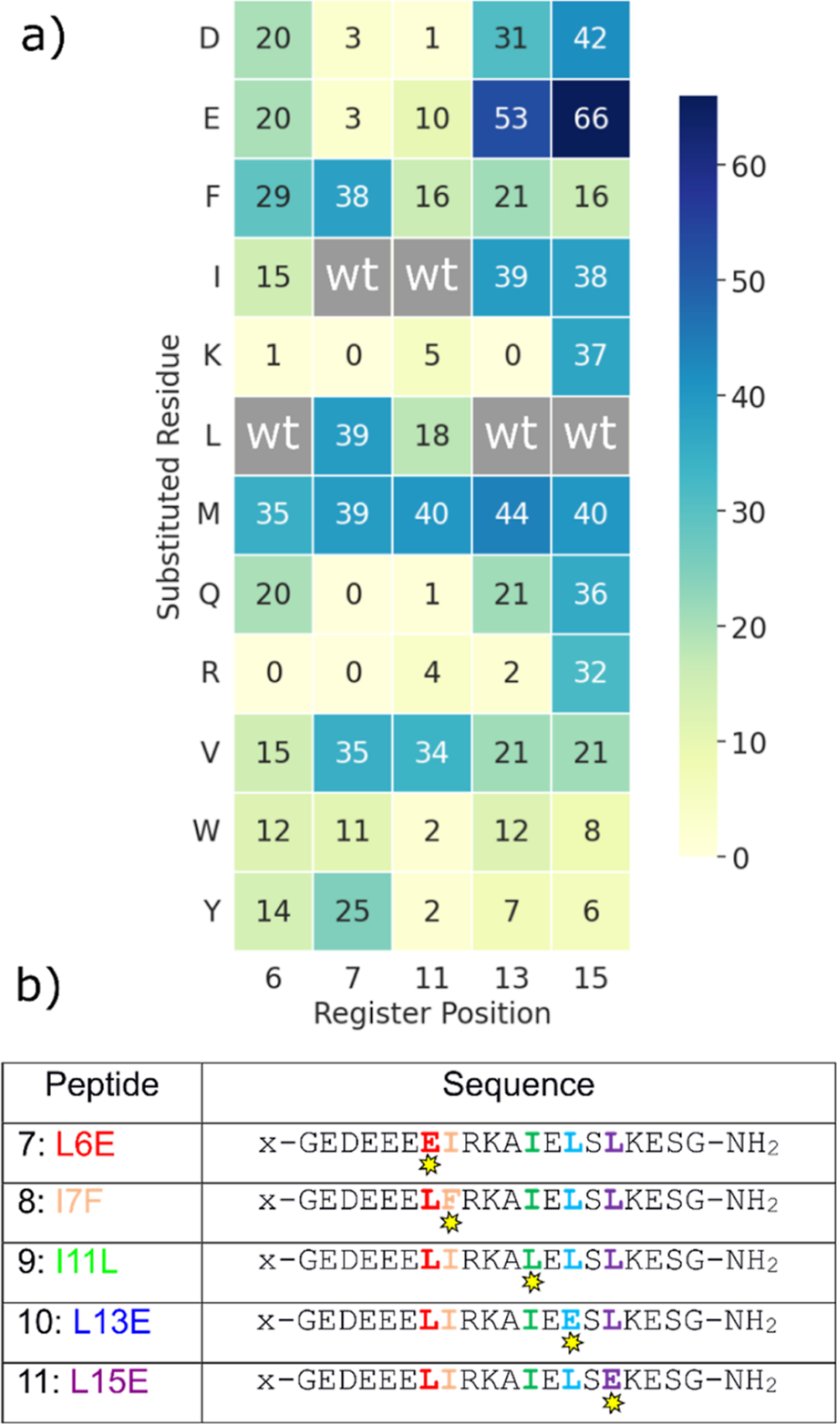
(a) Heatmap of displacement assay against the UIM-1 sequence, showing the number of models (out of 100 total) in which the UIM-1 sequence is displaced by the mutant peptide. Gray cells marked “wt” represent a match to the original UIM-1 sequence and were not screened. (b) UIM-1 variants studied experimentally x = FITC-β-alanyl (“**a**” series) or Ac-β-alanyl (“**b**” series).

During the course of our alanine scan experiments, we additionally carried out competition studies between the individual alanine mutants and the parent UIM-1 sequence. The UIM-1 sequence was found to be the stronger binder in all cases. Interestingly, the number of competing models occupied by the alanine mutant qualitatively followed the experimentally observed binding energies (Supporting Information, Table S9). This suggests that the competition experiments could be used to directly assess the potential of one sequence to bind more strongly than another and that the number of competing models may scale with the difference in binding energy. We, therefore, explored the use of AlphaFold Multimer competition experiments to guide the design of new binders. In order to select a set of residues to screen, we examined the frequency of amino acid occurrence in UIMs found in the SMART database^22,23^ as per the analysis by Lambrughi et al.^14^ These data are visualized in the WebLogo frequency plot (Supporting Information, Figure S4), in which the size of an amino acid single letter abbreviation reflects how frequently a particular residue is observed.^24^ We selected the residues D, E, F, K, I, L, M, Q, R, V, W, and Y, which are consistent with the sequence variation observed in UIM sequences. For completeness, each residue was screened at positions 6, 7, 11, 13, and 15 of the UIM-1 sequence. Each variant was competed against the parent UIM-1 sequence in a series of pairwise comparisons, and the number of models in which the mutant displaced the parent UIM-1 sequence was used as a measure of potential binding affinity. As before, the two sequences were modeled 100 times against both the Ub-decoy and decoy-Ub targets, and the top 50 models according to the AlphaFold multimer metric were analyzed (Figures 5a and S5).

In the vast majority of cases, the parent UIM-1 sequence was predicted by this screen to be the stronger binder. This is consistent with UIM-1 being a moderately high affinity UIM sequence and suggests that single-residue modification will generally destabilize the UIM-1:Ub interaction.^11,13,16,25^ In our study, only the L13E and L15E mutants were found to displace the parent sequence the majority of the time (53 and 66 models out of 100, respectively). Some other variants exhibited significant levels of displacement of the parent UIM-1 sequence, but none exceeded the 50% displacement level that would indicate a potential stronger binder.

There is a good overall agreement between our displacement screen and the analysis reported by Lambrughi et al.^14^ For example, basic residues are only observed to a significant extent at position 15 in the UIM register used here, and this behavior is recapitulated in our data. Similarly, glutamic acid residues are found to occur at positions 6, 13, and 15, but not at positions 7 or 11, and this tendency is again reflected in our data. Methionine is apparently favored at all positions in our displacement screen data but is never observed to outcompete the UIM-1 sequence. We have not yet studied any Met variants, and it remains an open question whether the competition screen could be perturbed by the high side-chain flexibility present in Met, but it is potentially not represented in the crystal structure data on which AlphaFold Multimer is trained.

**Figure 6:**
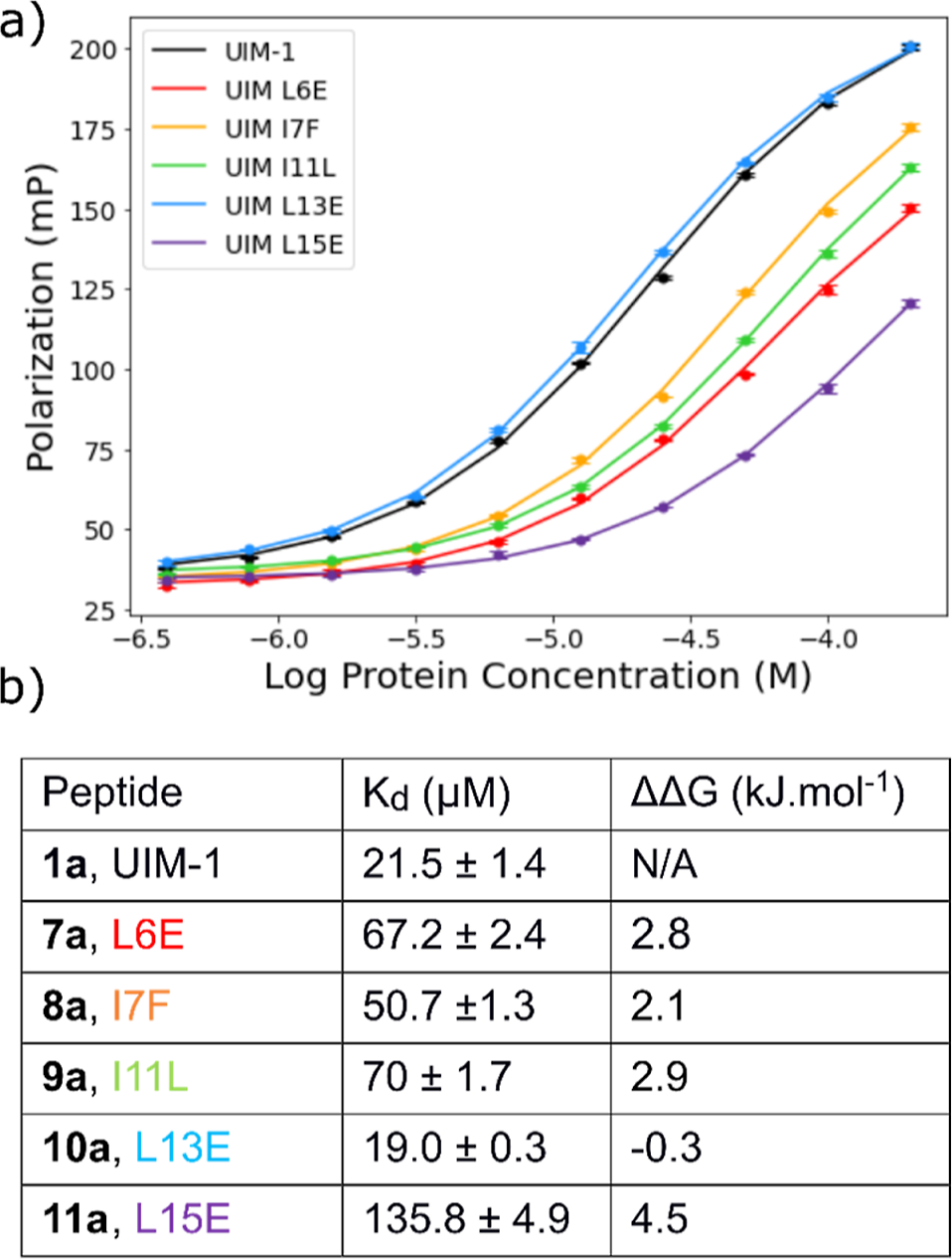
(a) FP titration curves for peptides **1a** and **7a–11a** against Ub. (b) *K*_d_ and ΔΔ*G* values for binding Ub. All experiments were for *N*-terminally-labeled peptides. Titrations were conducted in triplicate.

Based on the competition experiments, the L13E and L15E mutants were selected for experimental evaluation. Though it was not predicted to bind strongly, we also evaluated the L6E mutant as another variant with a comparable Leu to Glu change, which is strongly represented in UIMs. We additionally synthesized the I7F and I11L variants in which one of the two hydrophobic residues which contributes most to binding is altered. These peptides were synthesized and *N*-terminally fluorescein labeled as before, and FP titrations against Ub were carried out. The results of these titrations are shown in Figure 6:

Peptides **7a**–**9a** and **11a** were found to bind less strongly than the parent UIM-1 peptide **1a**. Peptide **10a** was bound marginally more strongly than **1a**, with a *K*_d_ value of 19.0 ± 0.3 μmol·L^–1^, equivalent to 0.3 kJ mol^–1^ additional binding energy. Importantly, the AlphaFold Multimer competition experiments correctly predicted the outcome in all but one of the titrations. L15E mutant **11a** was predicted by the competition experiments to be a stronger binder than the parent UIM1 sequence, but experimentally was found to bind more weakly than **1a**. Investigating the AlphaFold competition results more closely reveals that there is a significant bias between the results for the L15E mutant for the two different permutations of the Ub and decoy sequences. Specifically, the Ub-decoy sequence, in which the decoy fold is at the *C*-terminal end, gave the L15E variant bound in 24 out of 50 models. However, the decoy-Ub sequence (with the decoy at the N-terminal side) resulted in the L15E mutant being in the binding site on 42 out of 50 models (Figure S5). In all models, the Glu15 of **11a** and the binding site Ile/Ser 44 of the Ub/decoy Ub fold are separated by ∼10 Å, suggesting that the overprediction is not due to direct interactions between the peptide and either binding site. It is therefore not apparent why the decoy-Ub model overpredicts the binding of **11a**.

We compared the difference in results for the decoy-Ub and Ub-decoy competition experiments and found that there was generally a much larger difference in the results for positions 6 and 15, suggesting that end-effects can have a significant influence on the outcome of the competition experiments. When compared to the experimental data for the alanine scans and for the other mutants screened, neither individual ordering of Ub and decoy binding site gives better correlation with our experimental data than the combined data for both runs (Tables S7–S9 and Figure S5). This suggests that systematic errors can be eliminated to a large degree by taking both sets of models into account. Nevertheless, the large apparent preference for the L15E mutant is not entirely canceled out by this protocol, giving rise to an erroneous prediction of the stability of that sequence. However, the AlphaFold Multimer competition studies correctly predict the L13E mutant to be a stronger binder than the original UIM-1 sequence. Furthermore, when we conducted a further pairwise comparison between each binary pairing of the peptides UIM-1, L6E, I7F, I11L, L13E, and L15E, the correct ordering of binding strength was reproduced with the exception of the overprediction of the stability of L15E (Table S12), indicating that the competition assay results are self-consistent, despite the over-ranking of the L15E variant.

## Conclusions

In conclusion, we investigated the Ub:UIM-1 binding pair using a combined computational and experimental approach. We have demonstrated that it is possible to recapitulate the experimentally observed order of binding affinities for Ub:UIM-1 alanine mutants using AlphaFold Multimer competition experiments. Making a large number of models allows us to treat these competition experiments as a statistical rather than absolute measure of binding preference. These methods also reproduce experimentally observed data for MDM2/MDMX binding peptides. The use of a decoy protein fold, in which the protein target is duplicated but with a compromised binding site, can be used to prevent confounding peptide:peptide interactions in AlphaFold models, and this may be of particular value for low-affinity interactions, such as Ub:UIM-1. We further show that competition experiments of this type can be used as a means of refining the search for higher-affinity peptide binders. These studies demonstrate that it is possible to predict and control the Ub affinity of UIM sequences, and we anticipate that this will enable the design of customized Ub binding sequences as a tool in chemical biology. More generally, our studies show that AlphaFold Multimer competition experiments can be an accessible tool for peptide binder optimization and can distinguish small differences in binding strength arising from single residue sequence differences. We believe that this will allow the design of peptide binders with a tailored binding affinity for applications in chemical biology and beyond.

## Supporting Information Available

Full experimental methods and characterization for all peptides in this study and tabulated AlphaFold competition data (PDF)
