# Supplementary material for: AlphaFold Ensemble Competition Screens Enable Peptide Binder Design with Single-Residue Sensitivity
Source: ACS Chem Biol. 2024 Sep 18;19(10):2198–205. doi: 10.1021/acschembio.4c00418 (PMC11494501; doi:10.1021/acschembio.4c00418)
Supplement: Supplementary file 1 — cb4c00418_si_001.pdf [file cb4c00418_si_001.pdf]

Supporting Information

AlphaFold ensemble competition screens enable peptide binder design with single-residue sensitivity.

Pernille Vosbein[1], Paula Paredes Vergara[2], Danny T Huang[2,3], Andrew R Thomson[1,*]

Affiliations

[1]School of Chemistry, University of Glasgow, Glasgow G12 8QQ, UK

[2]Cancer Research UK Scotland Institute, Garscube Estate, Switchback Road, Glasgow, G61 1BD, UK

[3]School of Cancer Sciences, University of Glasgow, Glasgow, G61 1QH, UK

*Correspondence: Drew.Thomson@glasgow.ac.uk

Materials and Methods

General analytical procedures

Mass spectrometry was performed on a Bruker microTOF-Q High Resolution Mass Spectrometer using ESI+ mode.

Analytical RT-HPLC were performed on a Shimadzu reverse-phase HPLC system, equipped with Shimadzu LC-20AT pumps, a Shimadzu SIL-20A autosampler and a Shimadzu SPD-20A UV-Vis detector monitoring at 214 nm and 280 nm. The system was equipped with a Phenomenex, Aeris, 5 µm, peptide XB-C18, 150 x 4.6 mm column and run at a flow rate of 1 mL/min. Gradients were run using a binary solvent system consisting of solvent A (H_2O +0.1% TFA) and solvent B (MeCN + 0.1 TFA). Analytical HPLC data are reported as column retention time (RT) in minutes (min) as well as with the gradient.

LC-MS analysis was performed on an Agilent Infinity Lab liquid chromatography mass spectrometer, using positive mode electrospray ionization (ESI+), fitted with either a Shim-pack XR-ODS, C18, 2.2 µm, 2.0 x 50 mm column or a Gemini 5µm C18 110 Å, 150x2 mm LC column. The gradients were run at 0.3 mL/min flow rate using a binary solvent system of buffer A (H_2O + 0.1% TFA), and buffer B (MeCN + 0.1% TFA) over 20 mins.

In accordance with best practice for large molecules accurate mass spectra were not determined and masses are reported to one decimal place.[1,2]

Peptides were purified on a reverse-phase Dionex HPLC system equipped with Dionex P680 pumps and a Dionex UVD170U UV-vis detector (monitoring at 214 nm and 280 nm), using a Phenomenex, Gemini, C18, 5 µm, 250 x 21.2 mm column at a flow rate of 8 ml/min. Gradients were run using a solvent system consisting of A (H_2O + 0.1 % TFA) and B (MeCN + 0.1 % TFA). Collected fractions were lyophilized on a Christ Alpha 2-4 LO plus freeze dryer.

General procedure for peptide synthesis

Peptides were synthesized using a CEM Liberty Blue peptide synthesizer, using DIC/Oxyma activation and 20% morpholine in DMF for Fmoc deprotection. Peptides were synthesized on a 0.1 mmol scale using Rink amide polystyrene resins. Peptides were cleaved from the solid support using a cocktail of TFA/TIPS/water in 95:2.5:2.5 proportions. Peptides were precipitated by dilution in ice cold diethyl ether and recovered by centrifugation, then redissolved in 1:1 water/acetonitrile and freeze dried.

***N*-terminally labelled fluorescein peptides 1a-11a and 1c**

0.025 mmol of resin was used for fluorescein labelling reaction. Fluorescein isothiocyanate isomer 1 (3 equiv.) and DIPEA (3 equiv.) in DMF was added to the resin and reacted for 18 h at room temperature. The resin was washed with 5 mL DMF and three times with 5 mL DCM. Peptides were cleaved using the standard cleaving procedure. After purification *via* RT-HPLC and freeze drying, the peptides were obtained as yellow solids. Characterization data found in Table S1.

Capped peptides 1b-11b

0.025 mmol of resin was capped using acetic anhydride (50 equiv.) and pyridine (80 equiv.) in DMF. Reaction was carried out over 1h at room temperature. The resin was then washed with 5 mL DMF followed by 3 x 5mL washes with DCM. The peptides were cleaved using the standard cleaving procedure. After purification *via* RT-HPLC and lyophilization, the peptides were obtained as white solids. Characterization data found in Table S2.

Peptide 1c
0.025 mmol of the resin was capped using acetic anhydride (50 equiv.) and pyridine (80 equiv.) in DMF. The reaction was carried out over 1h at room temperature. The Alloc group was removed using $Pd(PPh_3)_4$ (0.25 equiv.) and morpholine (48 equiv.) in dry DCM for 2 h at room temperature. The peptide was fluorescein labelled using the same procedure as for the remaining fluorescein labelled peptides. Characterization data are given in Table S1

Protein Expression
Ub was expressed and purified as described previously.[3] Briefly, Ub was cloned into pRSFDuet vector (Novagen) containing a N-terminal hexahistidine tag followed by a TEV cleavage site and a Gly-Gly-Ser linker. Ub was expressed in *E. coli* Rosetta (DE3) cells. Cells were grown in Luria Bertani at 37° C and 0.2 mM isopropyl β-D-1 thiogalactopyranoside was added when OD600 reached 0.6-0.8. The cells were then grown overnight at 23° C. Cells were harvested in 25 mM Tris-HCl, pH 7.6, 0.15 M NaCl, 20 mM imidazole, 2.5 mM phenylmethanesulfonyl fluoride and 5 mM β-mercaptoethanol and lysed by a microfluidizer. The clarified lysates were applied onto Ni^{2+}-affinity column, washed with 25 mM Tris-HCl, pH 7.6, 0.15 M NaCl, 20 mM imidazole and 5 mM β-mercaptoethanol, and then eluted with 25 mM Tris-HCl, pH 7.6, 0.15 M NaCl, 200 mM imidazole and 5 mM β-mercaptoethanol. His-tag Ub was cleaved with TEV protease and dialyzed against buffer containing 25 mM Tris-HCl, pH 7.6, 0.15 M NaCl and 5 mM β-mercaptoethanol at room temperature overnight. The cleaved Ub was subjected to Ni^{2+}-affinity column pass-back to remove any uncleaved Ub. The flow through containing cleaved Ub was further purified by gel filtration chromatography with SD75 1660 column in buffer containing 25 mM Tris-HCl, pH 7.6, 0.15 M NaCl and 1 mM DTT. Ub concentration was determined by using absorbance at 280 nm and the calculated molar extinction coefficient.

Peptides	Sequence	Purification gradient	Purity HPLC	HRMS
**1a**	FITC-βA-GEDEEELIRKAIELSLKESG-NH_2	24-70% B	35.96 min 100% (50 min gradient, 20-50% B).	Calculated for $C_{119}H_{178}N_{28}O_{42}S$ $[M+H4]^{4+}$ 2705.3 found 2705.1
**1c**	Ac-GEDEEELIRKAIELSLKESGK(FITC)-NH_2	35-60% B	31.46 min 93.99% (50 min gradient, 20-60% B)	Calculated for $C_{124}H_{187}N_{29}O_{43}S$ $[M+H2]^{2+}$ 2804.3 found 2803.3
**2a**	FITC-βA-GEDEEEAIRKAIELSLKESG-NH_2	30-55% B	15.90 min 95.55% (50 min gradient, 30-60% B).	Calculated for $C_{116}H_{172}N_{28}O_{42}S$ $[M+H2]^{2+}$ 2664.2 found 2664.2
**3a**	FITC-βA-GEDEEELARKAIELSLKESG-NH_2	30-50% B	13.80 min 93.89% (50 min gradient, 30-60% B).	Calculated for $C_{97}H_{163}N_{27}O_{38}$ $[M+H2]^{2+}$ 2317.2 found 2317.2
**4a**	FITC-βA-GEDEEELIRKAAELSLKESG-NH_2	30-45% B	12.58 min 93.67% (50 min gradient, 30-60% B).	Calculated for $C_{116}H_{172}N_{28}O_{42}S$ $[M+H2]^{2+}$ 2664.2 found 2664.1
**5a**	FITC-βA-GEDEEELIRKAIEASLKESG-NH_2	30-45% B	16.56 min 96.33% (50 min gradient, 30-60% B).	Calculated for $C_{116}H_{172}N_{28}O_{42}S$ $[M+H2]^{2+}$ 2664.2 found 2664.1
**6a**	FITC-βA-GEDEEELIRKAIELSAKESG-NH_2	30-50% B	14.89 min 91.94% (50 min gradient, 30-60% B).	Calculated for $C_{116}H_{172}N_{28}O_{42}S$ $[M+H2]^{2+}$ 2664.2 found 2664.1
**7a**	FITC-βA-GEDEEEEIRKAIELSLKESG-NH_2	25-55% B	32.39 min 100% (50 min gradient, 20-50% B).	Calculated for $C_{118}H_{174}N_{28}O_{44}S$ $[M+H2]^{2+}$ 2721.2 found 2721.1
**8a**	FITC-βA-GEDEEELFRKAIELSLKESG-NH_2	30-60% B	35.17 min 100% (50 min gradient, 20-50% B).	Calculated for $C_{119}H_{178}N_{28}O_{42}S$ $[M+H2]^{2+}$ 2705.3 found 2705.2
**9a**	FITC-βA-GEDEEELIRKALELSLKESG-NH_2	25-55% B	18.14 min 97.02% (50 min gradient, 30-60% B).	Calculated for $C_{122}H_{176}N_{28}O_{42}S$ $[M+H2]^{2+}$ 2739.2 found 2739.0
**10a**	FITC-βA-GEDEEELIRKAIEESLKESG-NH_2	25-55% B	33.63 min 90.59% (50 min gradient, 20-50% B).	Calculated for $C_{118}H_{174}N_{28}O_{44}S$ $[M+H3]^{3+}$ 2721.2 found 2721.1
**11a**	FITC-βA-GEDEEELIRKAIELSEKESG-NH_2	30-55% B	32.62 min 96.25% (50 min gradient, 20-50% B).	Calculated for $C_{118}H_{174}N_{28}O_{44}S$ $[M+H3]^{3+}$ 2722.2 found 2722.2

Table S1: Peptide sequence and characterization data for peptides **1a** - **11a**.

Peptides	Sequence	Purification gradient	Purity HPLC	HRMS
**1b**	Ac-GEDEEELIRKAIELSLKESG-NH_2	25-70% B	13.53 min 95.68% (50 min gradient, 30-60% B).	Calculated for $C_{97}H_{164}N_{26}O_{37}$ $[M+H2]^{2+}$ 2287.2 found 2287.2
**2b**	Ac-βA-GEDEEEAIRKAIELSLKESG-NH_2	30-50% B	26.89 min 98.43% (50 min gradient, 20-50% B).	Calculated for $C_{97}H_{163}N_{27}O_{38}$ $[M+H2]^{2+}$ 2316.2 found 2316.2
**3b**	Ac-βA-GEDEEELARKAIELSLKESG-NH_2	30-50% B	24.39 min 89.50% (50 min gradient, 20-50% B).	Calculated for $C_{97}H_{163}N_{27}O_{38}$ $[M+H2]^{2+}$ 2316.2 found 2316.2
**4b**	Ac-βA-GEDEEELIRKAAELSLKESG-NH_2	30-45% B	23.26 min 96.38% (50 min gradient, 20-50% B).	Calculated for $C_{97}H_{163}N_{27}O_{38}$ $[M+H2]^{2+}$ 2316.2 found 2316.2
**5b**	Ac-βA-GEDEEELIRKAIEASLKESG-NH_2	25-45% B	27.75 min 96.79% (50 min gradient, 20-50% B).	Calculated for $C_{97}H_{163}N_{27}O_{38}$ $[M+H2]^{2+}$ 2316.2 found 2316.2
**6b**	Ac-βA-GEDEEELIRKAIELSAKESG-NH_2	25-45% B	25.73 min 93.72% (50 min gradient, 20-50% B).	Calculated for $C_{97}H_{163}N_{27}O_{38}$ $[M+H2]^{2+}$ 2316.2 found 2316.2
**7b**	Ac-βA-GEDEEEEIRKAIELSLKESG-NH_2	25-50% B	26.36 min 95.04% (50 min gradient, 20-50% B).	Calculated for $C_{99}H_{165}N_{27}O_{40}$ $[M+H2]^{2+}$ 2376.2 found 2377.5
**8b**	Ac-βA-GEDEEELFRKAIELSLKESG-NH_2	30-60% B	28.97 min 100% (50 min gradient, 30-60% B).	Calculated for $C_{100}H_{169}N_{27}O_{38}$ $[M+H2]^{2+}$ 2358.2 found 2358.2
**9b**	Ac-βA-GEDEEELIRKALELSLKESG-NH_2	30-55% B	28.96 min 100% (50 min gradient, 20-50% B).	Calculated for $C_{103}H_{167}N_{27}O_{38}$ $[M+H2]^{2+}$ 2392.2 found 2392.2
**10b**	Ac-βA-GEDEEELIRKAIEESLKESG-NH_2	25-60% B	27.37 min 100% (50 min gradient, 20-50% B).	Calculated for $C_{99}H_{165}N_{27}O_{40}$ $[M+H2]^{2+}$ 2374.2 found 2374.1
**11b**	Ac-βA-GEDEEELIRKAIELSEKESG-NH_2	25-50% B	26.24 min 92.23% (50 min gradient, 20-50% B)	Calculated for $C_{99}H_{165}N_{27}O_{40}$ $[M+H2]^{2+}$ 2374.2 found 2374.1

Table S2: Peptide sequence and characterization data for peptides **1b – 11b**

FP Assays

Fluorescence polarization assays were preformed using a BMG Labtech CLARIOstar plate reader with monochrome filters measuring excitation wavelengths of 482-16 nm and emission wavelength of 530-40 nm. The assays were run using the top optics, a settling time of 0.5 s and 500 flashes per well. Assays were run in Corning 384 well, black, low volume, round bottom polystyrene non-binding surface well plates. All titration curves were measured in triplicate and error bars on plotted graphs show +/- one standard deviation.

FP assays were performed based on the basic protocol by Moerke in 2009.[4] The assays were run in MES buffer consisting of 0.2 g MES w/v, 0.09 g w/v and 0.05% Tween 20 in H_2O. A stock solution of the protein of interest was prepared by adding the appropriate amount of protein to an Eppendorf tube with 5 µL of 10x MES buffer. Distilled water was added to the Eppendorf tub to a total volume of 50 µL. A 10 point 1:2 serial dilution of the protein was performed by sequentially mixing 25 µL of 1xMES buffer with 25 µL of the previous protein solution in a new Eppendorf tube. 25 µL of a 100 nM stock of the fluorescently labelled peptide in 1xMES buffer was added to each of the protein dilution tubes for a final concentration of 50 nM labelled peptide. 10 µL of protein dilution/labelled peptide solution was transferred to a black opaque 384-well plate in triplicate. 3x10 µL of 1xMES buffer was also transferred for background measurements as well as 3x10 µL of a 50 nM labelled peptide solution for gain adjustment. The plate was added to the plate reader and the gain was adjusted to 30 mP using the well with the 50 nM labelled peptide solution. The plate was measured, and the results were exported to a .csv file. The data was corrected for background contributions from the buffer by subtracting the values from the wells containing the buffer. Data were fitted to a 1:1 binding model using the 'PyBindingCurve' Python package (https://stevenshave.github.io/pybindingcurve/)

CD Measurements

Circular dichroism measurements were performed on an Applied Photophysics Chirascan VX circular dichroism spectropolarimeter, equipped with a Quantum Northwest TC Temperature controller and a CW-3000 industrial chiller, or on a JASCO J-810 circular dichroism spectropolarimeter equipped with a Peltier sample stage. CD spectra were recorded in MOPS buffer at pH 7, concentration of 50 µM and 20 °C unless otherwise stated. The HT signal stayed below 500 V to at least 180 nM unless otherwise stated.

General Computational

Models were run using colabfold local v1.5.5 (https://github.com/YoshitakaMo/localcolabfold) on a workstation equipped with an RTX4080 GPU.[5]

AlphaFold Ensemble Competition Experiments

All competition experiments were carried out as a binary competition between two potential peptide binders and a protein target. In the case of Ub this was either a single Ub domain, or two fused Ub domains, one of which was a 'decoy' with the I44S mutation at the binding site. MDM2 and MDMX were competed individually against the pMI ligand as single domains. Competition experiments were carried out by generating 100 models, incrementing through 100 numerical seeds from a randomly generated five-digit seed. For each analysis the top 50 models according to the AlphaFold Multimer metric were analyzed. In the case of the two configurations of the Ub-decoy or decoy-Ub chains this gave 100 models in total.

Peptide binders were identified using a custom python script using the 'Isambard' module (https://github.com/isambard-uob/isambard). The distance between key residues in the

peptide binder and protein domain were measured. In the case of the Ub/decoy systems only models in which both peptides were engaged with either the Ub or decoy were considered: the small numbers (typically 0 – 2, max 3) of models per 100 model run in which one peptide strand was not engaged with the decoy domain were removed from the analysis.

CD Data

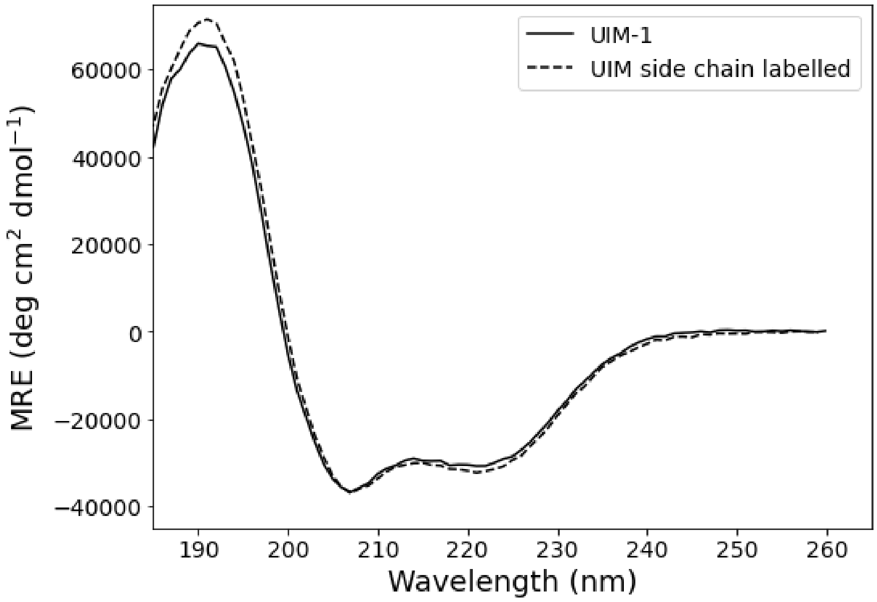

Figure S1: CD spectra for **1a** (solid line) and **1b** (dashed line).

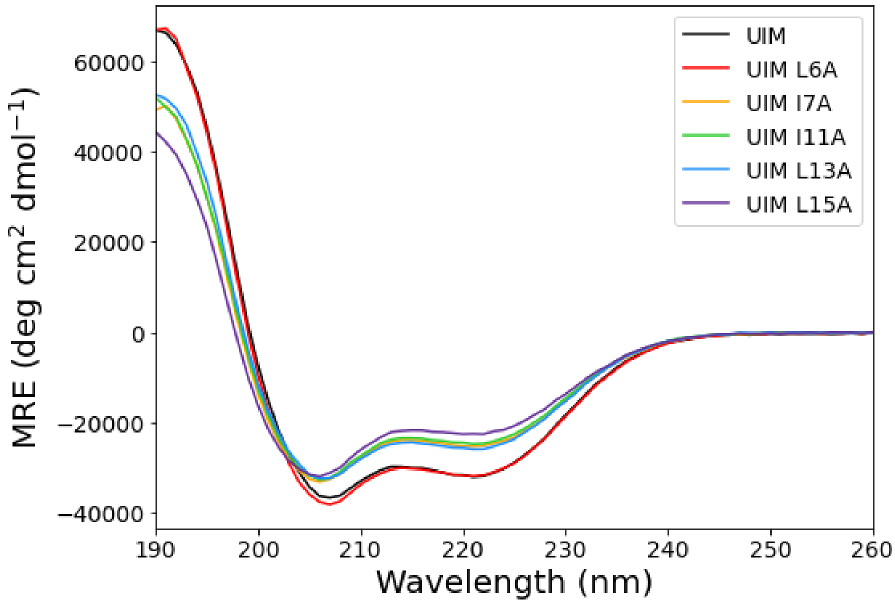

Figure S2: CD spectra for **1b** (black line), **2b** (red line), **3b** (orange line), **4b** (green line), **5b** (blue line) and **6b** (purple line).

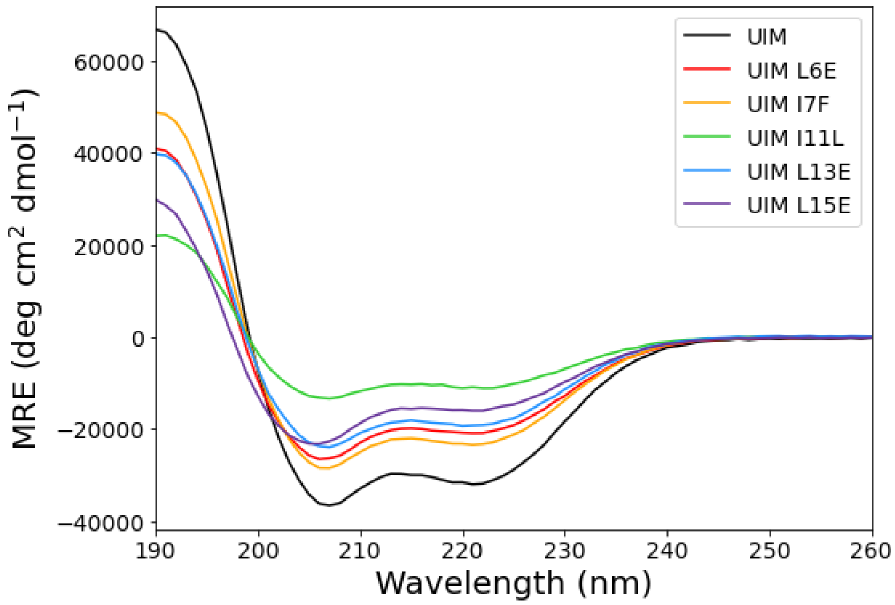

Figure S3: CD spectra for **1b** (black line), **7b** (red line), **8b** (orange line), **9b** (green line), **10b** (blue line) and **11b** (purple line).

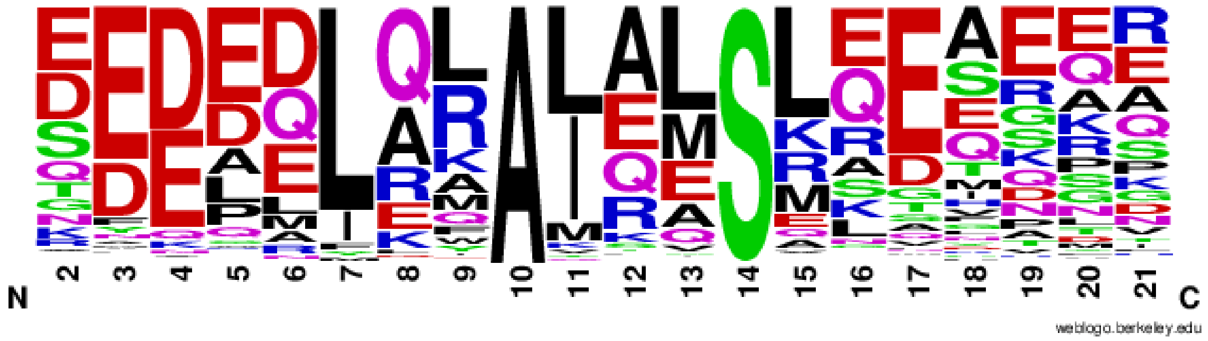

Figure S4: Frequency plot of UIM sequences from Lambrughi et al.[6] Residue numbering as for peptides 1 – **11**.

Outputs for Alanine Scanning and Mutation Experiments
Peptide residues shown in bold were individually mutated to alanine and all pairwise combinations were subject to AlphaFold competition experiments.

Ubiquitin:UIM-1 alanine scan (no decoy, 100 models, top 50 analyzed).

Ub
MQIFVKTLTGKTITLEVEPSDTIENVKAKIQDKEGIPPDQQRLIFAGKQLEDGRTLSDYNIQKEST
LHLVLRLRGG

UIM-1:
GEDEEE**LI**RKA**I**E**L**S**L**KESG

I7A	L6A (50)	x			
I11A	L6A (40)	I11A (48)	x		
L13A	L13A (26)	L13A (50)	L13A (40)	x	
L15A	L15A (36)	L15A (50)	L15A (47)	L15A (45)	x
wt	wt (36)	wt (50)	wt (50)	wt (38)	wt (50)
	L6A	I7A	I11A	L13A	L15A

Table S3. Alanine scan for pairwise competition of UIM-1 alanine mutants in binding Ub. Top binder shown for each comparison. Number of bound models shown in brackets. Data inconsistent with experimental values highlighted in red.

Gives ranking wt > I7 > I11 > L6 > L13 > L15 (experimentally wt > I7 > I11 > L15 > L6 > L13)

MDM2:pMI alanine scan (no decoy, 100 models, top 50 analyzed)
MDM2 sequence
QETLVRPKPLLLKLLKSVGAQKDTYTMKEVLFYLGQYIMTKRLYDEKQQHIVYCSNDLLGDLFGVP
SFSVKEHRKIYTMIYRNLVVVNQQ:

pMI
TS**F**AE**YW**NL**L**SP

Y6A	Y6A (50)	x	
W7A	F3A (47)	Y6A (50)	x
L10A	L10A (50)	Y6A (50)	L10A (50)
	F3A	Y6A	W7A

Table S4: Alanine scan for pMI peptide vs MDM2
Top binder shown for each comparison. Number of bound models shown in brackets.

Gives ranking W7 > F3 > L10 > Y6 (matches experimental ordering)

MDMX:pMI alanine scan (no decoy, 100 models, top 50 analyzed)

MDMX
QINQVRPKLPLLKILHAAGAQGEMFTVKEVMHYLGQYIMVKQLYDQQEQHMVYCGGDLLGELLGRQ
SFSVKDPSPLYDMLRKNLVTLAT

pMI
TS**F**AE**YW**NL**L**SP

Y6A	Y6A (50)	x	
W7A	F3A (50)	Y6A (50)	x
L10A	L10A (50)	L10A (32)	L10A (50)
	F3A	Y6A	W7A

Table S5: Alanine scan for pMI peptide vs MDMX. Top binder shown for each comparison. Number of bound models shown in brackets.

Gives ranking W7 > F3 > Y6 > L10 (matches experimental ordering)

I44ADecoy-Ub:UIM-1 alanine scan (100 models, top 50 analyzed).

Decoy-Ub
MQIFVKTLTGKTITLEVEPSDTIENVKAKIQDKEGIPPDQQRL**A**FAGKQLEDGRTLSDYNIQKEST
LHLVLRLRGGGGMQIFVKTLTGKTITLEVEPSDTIENVKAKIQDKEGIPPDQQRL**I**FAGKQLEDGR
TLSDYNIQKESTLHLVLRLRGG

UIM-1
GEDEEE**LI**RKA**I**E**L**S**L**KESG

I7A	L6A (48)	x			
I11A	L6A (26)	I11A (49)	x		
L13A	== (25 each)	L13A (47)	L13A (26)	x	
L15A	L6A (43)	L15A (35)	I11A (47)	L13A (41)	x
wt	L6A (34)	wt (38)	I11A (30)	L13A (32)	wt (33)
	L6A	I7A	I11A	L13A	L15A

Table S6: Alanine scan for pairwise competition of UIM-1 alanine mutants in binding I44ADecoy-Ub. Top binder shown for each comparison. Number of bound models shown in brackets. Data inconsistent with experimental values highlighted in red.

Gives ranking I7 > L15 > wt > I11 > L6 == L13

I44SDecoy-Ub:UIM-1 alanine scan (100 models, top 50 analyzed).

Decoy-Ub
MQIFVKTLTGKTITLEVEPSDTIENVKAKIQDKEGIPPDQQRLSFAGKQLEDGRTLSDYNIQKEST
LHLVLRLRGGGGMQIFVKTLTGKTITLEVEPSDTIENVKAKIQDKEGIPPDQQRLIFAGKQLEDGR
TLSDYNIQKESTLHLVLRLRGG

UIM-1
GEDEEE**LI**RKA**I**E**L**S**L**KESG

I7A	L6A (50)	x			
I11A	L6A (43)	I11A (49)	x		
L13A	L13A (27)	L13A (50)	L13A (44)	x	
L15A	L6A (32)	L15A (49)	L15A (40)	L13A (40)	x
wt	wt (30)	wt (49)	wt (42)	L13A (28)	wt (28)
	L6A	I7A	I11A	L13A	L15A

Table S7: Alanine scan for pairwise competition of UIM-1 alanine mutants in binding I44SDecoy-Ub. Top binder shown for each comparison. Number of bound models shown in brackets. Data inconsistent with experimental values highlighted in red.

Ub-I44Sdecoy:UIM-1 alanine scan (100 models, top 50 analyzed).

Ub-decoy
MQIFVKTLTGKTITLEVEPSDTIENVKAKIQDKEGIPPDQQRLIFAGKQLEDGRTLSDYNIQKEST
LHLVLRLRGGGGMQIFVKTLTGKTITLEVEPSDTIENVKAKIQDKEGIPPDQQRLSFAGKQLEDGR
TLSDYNIQKESTLHLVLRLRGG

UIM-1
GEDEEE**LI**RKA**I**E**L**S**L**KESG

I7A	L6A (49)	x			
I11A	L6A (47)	== (25 each)	x		
L13A	L13A (29)	L13A (49)	L13A (47)	x	
L15A	L6A (37)	L15A (41)	L15A (40)	L13A (37)	x
wt	wt (33)	wt (49)	wt (48)	wt (29)	wt (46)
	L6A	I7A	I11A	L13A	L15A

Table S8: Alanine scan for pairwise competition of UIM-1 alanine mutants in binding Ub-I44SDecoy. Top binder shown for each comparison. Number of bound models shown in brackets. Data inconsistent with experimental values highlighted in red.

Composite values for combined decoy scans

I7A	L6A (99)	x			
I11A	L6A (90)	I11A (74)	x		
L13A	L13A (56)	L13A (99)	L13A (91)	x	
L15A	L6A (69)	L15A (90)	L15A (80)	L13A (77)	x
wt	wt (63)	wt (98)	wt (90)	wt (51)	wt (74)
	L6A	I7A	I11A	L13A	L15A

Table S9: Aggregate values for data in tables S6 and S7

Gives ranking I7 > I11 > L15 > L6 > L13

Reproducibility of L13A-L15A Competition
Re-running the I44SDecoy-Ub and Ub-I44SDecoy screens for the L13A/L15A pair a further three times with different random seeds gave the L13A sequence bound to Ub in 82/100, 69/100 and 72/100 models. This gives an average of 76.25/100 with a standard deviation of 5.4.

MDM2-Decoy:pMI alanine scan (no decoy, 100 models, top 50 analyzed)

```
MDM2-decoy
QETLVRPKPLLLKLLKSVGAQKDTYTMKEVLFYLGQYIMTKRLYDEKQQHIVYCSNDLLGDLFGVP
SFSVKEHRKIYTMIYRNLVVVNQQGG
QETLVRPKPLLLKLLKSVGAQKDTYTMKEVSFYSGQYSMTKRLYDEKQQHIVYCSNDLLGDLFGVP
SFSSKEHRKSYTMIYRNLVVVNQQ
pMI
TSFAEYWNLLSP
```

Y6A	Y6A (46)	x	
W7A	F3A (44)	Y6A (50)	x
L10A	L10A (41)	Y6A (41)	L10A (50)
	F3A	Y6A	W7A

Table S10: Alanine scan for pMI peptide vs MDM2
Top binder shown for each comparison. Number of bound models shown in brackets.

Gives ranking W7 > F3 > L10 > Y6 (matches experimental ordering)

MDMX-Decoy:pMI alanine scan (no decoy, 100 models, top 50 analyzed)
MDMX
QINQVRPKLPLLKILHAAGAQGEMFTVKEVMHYLGQYIMVKQLYDQQEQHMVYCGGDLLGELLGRQ
SFSVKDPSPLYDMLRKNLVTLATGG
QINQVRPKLPLLKILHAAGAQGEMFTVKEVSHYSGQYSMVKQLYDQQEQHMVYCGGDLLGELLGRQ
SFSSKDPSPSYDMLRKNLVTLAT:
pMI
TS**F**AE**YW**NL**L**SP

Y6A	Y6A (50)	x	
W7A	F3A (44)	Y6A (50)	x
L10A	L10A (49)	Y6A (36)	L10A (50)
	F3A	Y6A	W7A

Table S11: Alanine scan for pMI peptide vs MDMX-Decoy. Top binder shown for each comparison. Number of bound models shown in brackets. Data inconsistent with experimental values highlighted in red.

Gives ranking W7 > F3 > L10 > Y6 (L10 and Y6 mis-ordered)

Competition of UIM-1 point mutants vs decoy-Ub and Ub-decoy (100 models, top 50 analyzed)

Highlighted residues in UIM-1 were altered to one of D, E, F, I, K, L, M, Q, R, V, W or Y, and the resulting sequence competed against UIM-1 in binding either decoy-Ub or Ub-decoy sequence.

Decoy-Ub
MQIFVKTLTGKTITLEVEPSDTIENVKAKIQDKEGIPPDQQRLSFAGKQLEDGRTLSDYNIQKEST
LHLVLRLRGGGGMQIFVKTLTGKTITLEVEPSDTIENVKAKIQDKEGIPPDQQRLIFAGKQLEDGR
TLSDYNIQKESTLHLVLRLRGG

Ub-decoy
MQIFVKTLTGKTITLEVEPSDTIENVKAKIQDKEGIPPDQQRLIFAGKQLEDGRTLSDYNIQKEST
LHLVLRLRGGGGMQIFVKTLTGKTITLEVEPSDTIENVKAKIQDKEGIPPDQQRLSFAGKQLEDGR
TLSDYNIQKESTLHLVLRLRGG

UIM-1
GEDEEE**LI**RKA**I**E**L**S**L**KESG

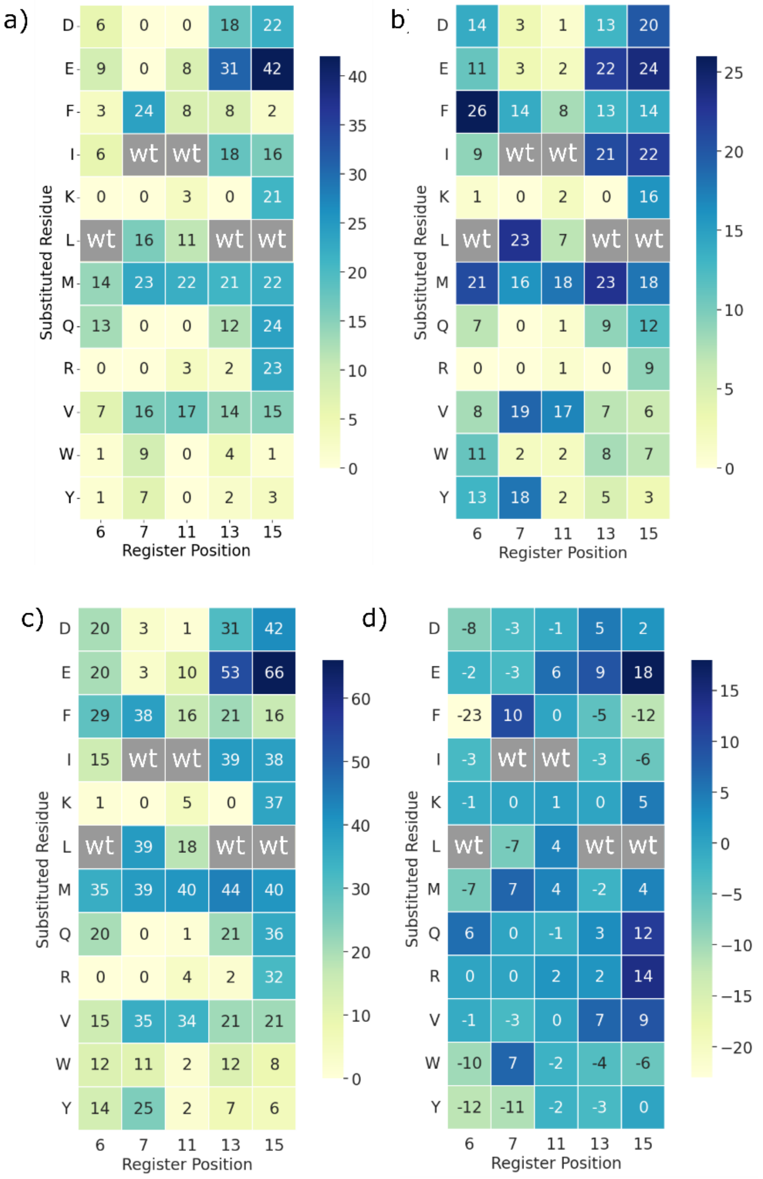

Figure S5: a) Heatmap for decoy-Ub point mutant screen b) Heatmap for Ub-decoy point mutant screen. c) Composite numbers for decoy-Ub and Ub-decoy point mutant screens and d) Difference heatmap showing difference in number of bound mutant sequences (decoy-Ub minus Ub decoy values). Cells marked with ‘wt’ correspond to a match with the UIM-1 sequence and comparisons were not run.

I7F	I7F (77)	x			
I11L	I11L (52)	I7F (56)	x		
L13E	L13E (87)	L13E (63)	L13E (75)	x	
L15E	L15E (86)	L15E (78)	L15E (87)	L15E (77)	x
wt	wt (80)	wt (97)	wt (90)	L13E (53)	wt (34)
	L6E	I7F	I11L	L13E	L15E

Table S12: Aggregate data for pairwise competition between point mutants of the UIM-1 sequence in binding decoy-Ub or Ub-decoy. Top binder shown for each comparison. Number of bound models shown in brackets. Data inconsistent with experimental values highlighted in red.

HPLC Characterization for all peptides

Peptide 1a

35.96 min 100% (50 min gradient, 20-50% B).

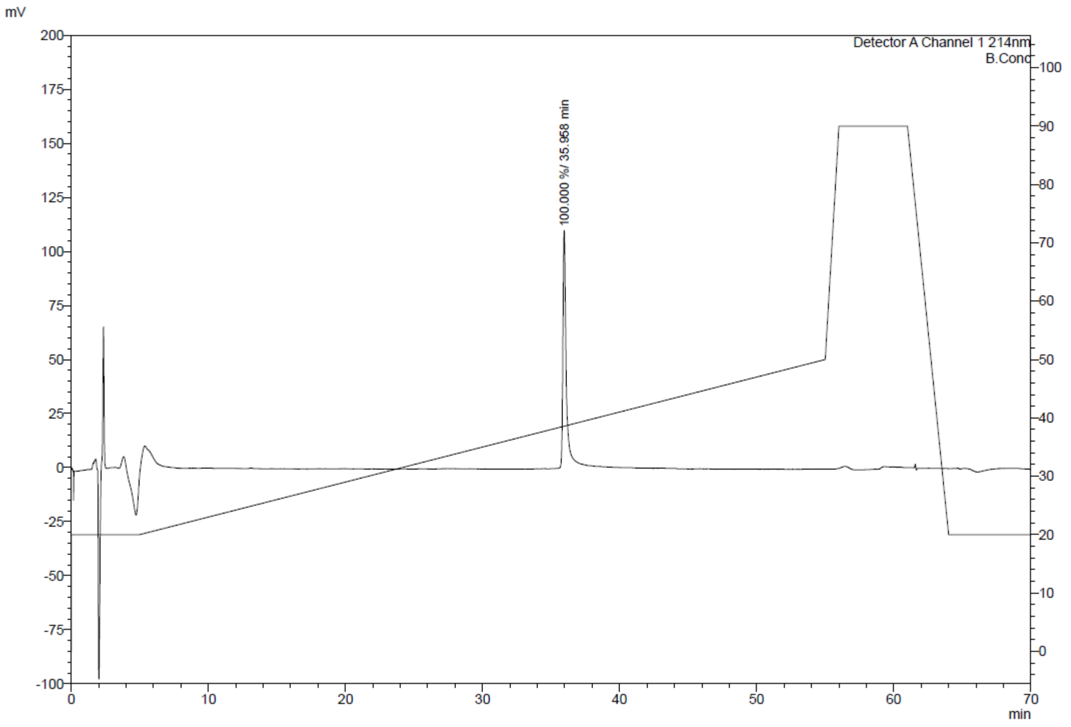

*SI Figure 1: 50 min gradient 20-50% B HPLC for **1a**.*

Peptide 1c

31.46 min 93.99% (50 min gradient, 20-60% B).

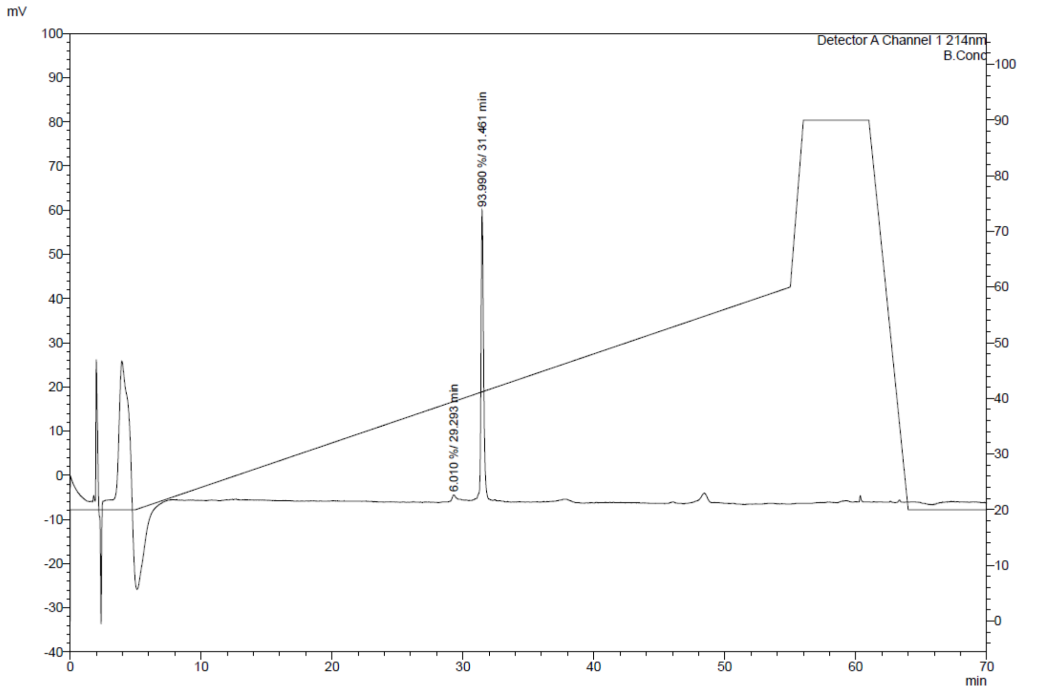

*SI Figure 2: 50 min gradient 20-60% B HPLC for peptide **1c**.*

Peptide 2a

15.90 min 95.55% (50 min gradient, 30-60% B).

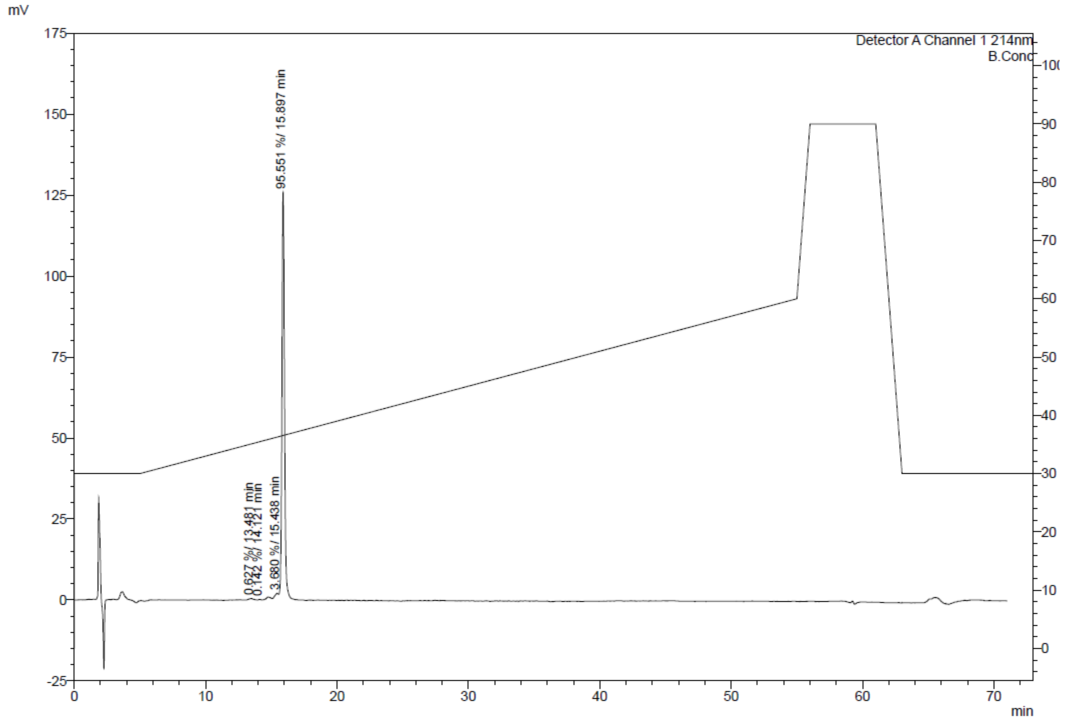

*SI Figure 3: 50 min gradient 30-60% B HPLC for peptide **2a**.*

Peptide 3a

13.80 min 93.89% (50 min gradient, 30-60% B).

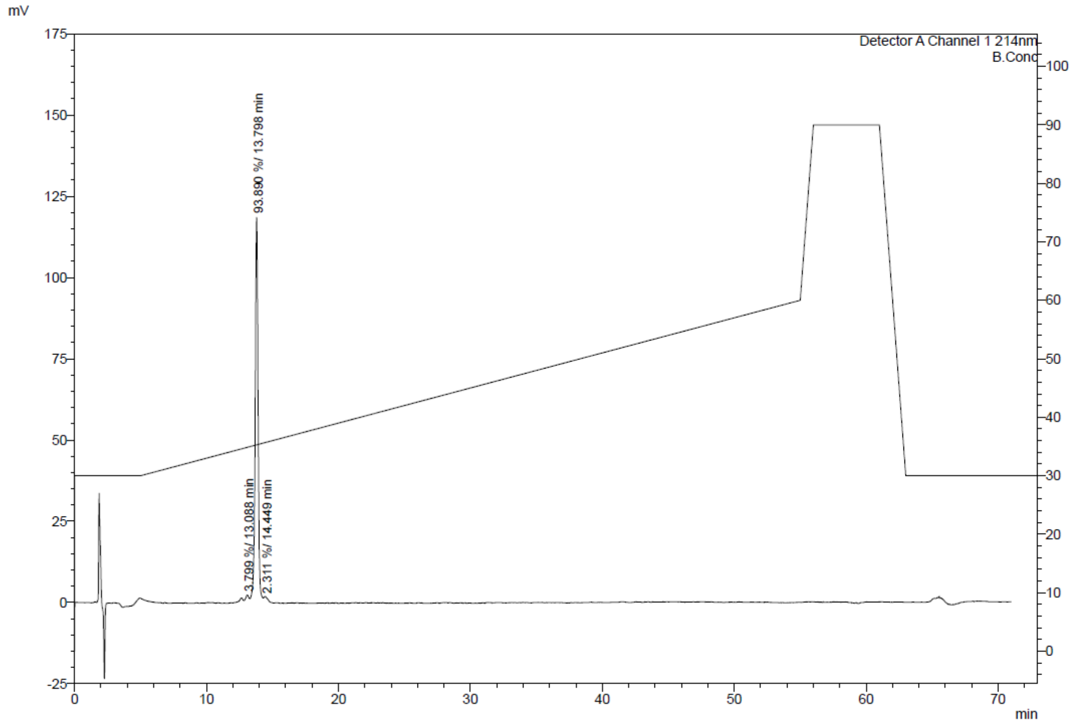

*SI Figure 4: 50 min gradient 30-60% B HPLC for peptide **3a**.*

Peptide 4a
12.58 min 93.67% (50 min gradient, 30-60% B).

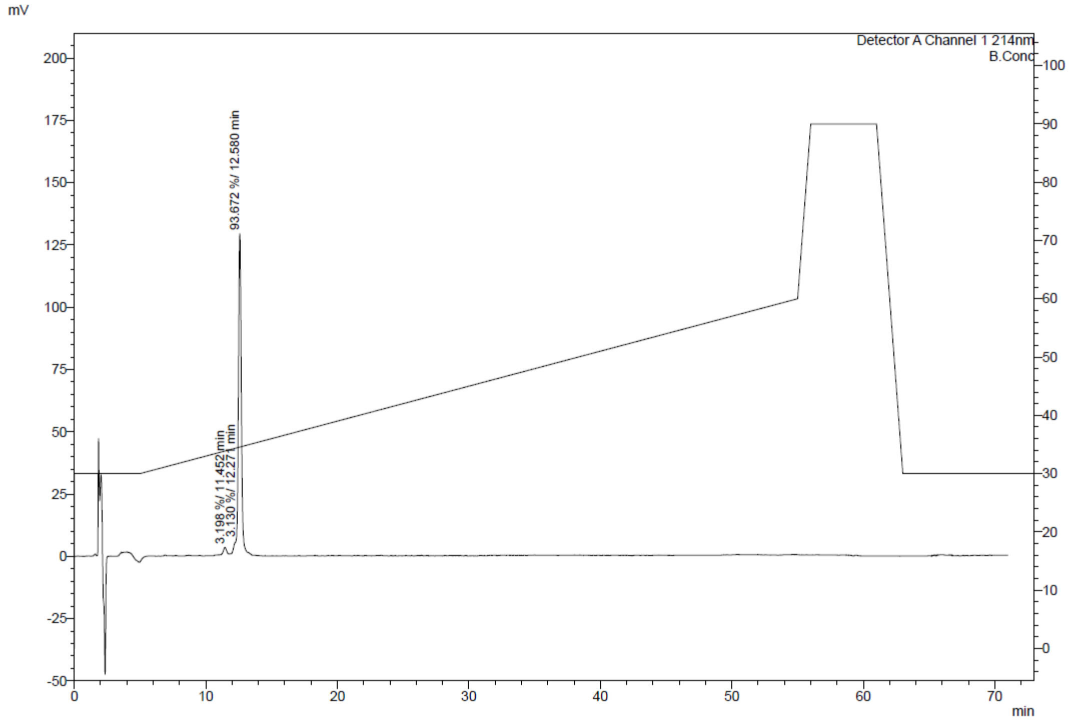

*SI Figure 5: 50 min gradient 30-60% B HPLC for peptide **4a***

Peptide 5a
16.56 min 96.33% (50 min gradient, 30-60% B).

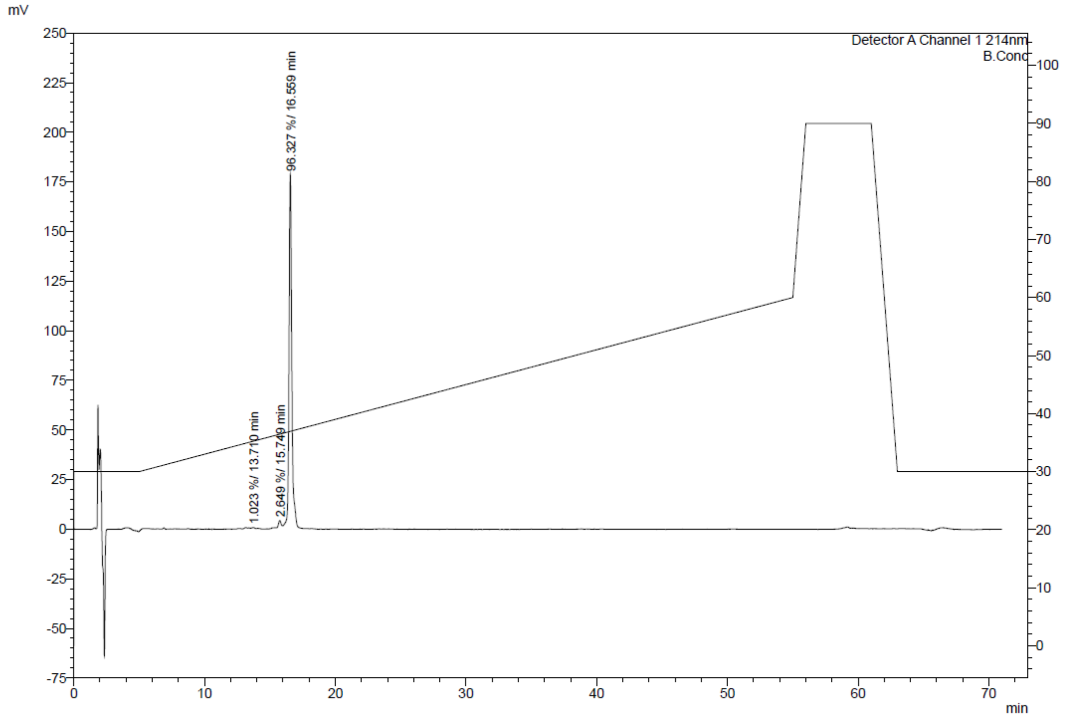

*SI Figure 6: 50 min gradient 30-60% B HPLC for peptide **5a**.*

Peptide 6a

14.89 min 91.94% (50 min gradient, 30-60% B).

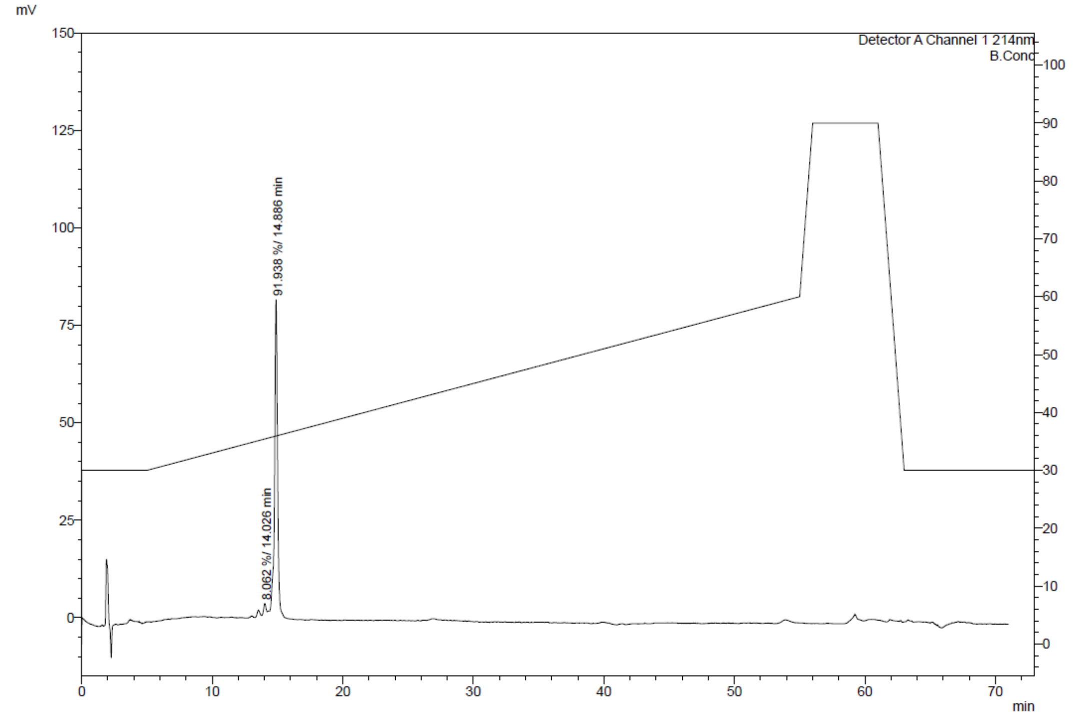

SI Figure 7: 50 min gradient 30-60% B HPLC for peptide ***6a****.*

Peptide 7a

32.39 min 100% (50 min gradient, 20-50% B).

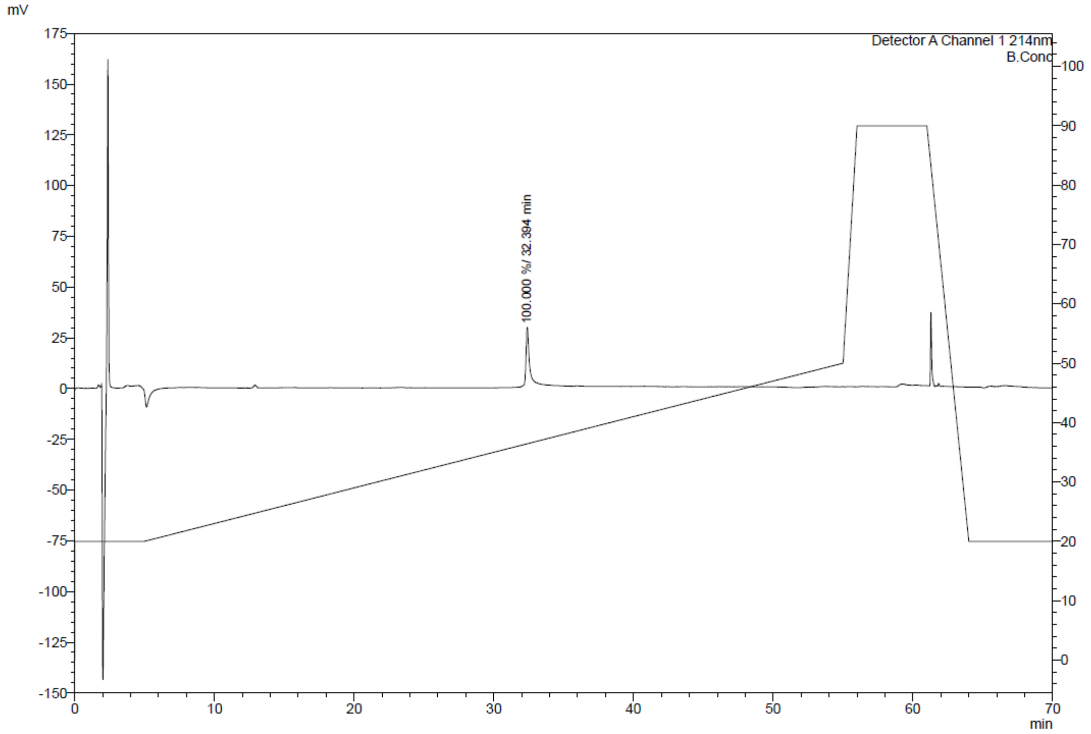

SI Figure 8: 50 min gradient 20-50% B HPLC for peptide ***7a****.*

Peptide 8a

33.628 min 90.59% (50 min gradient, 20-50% B).

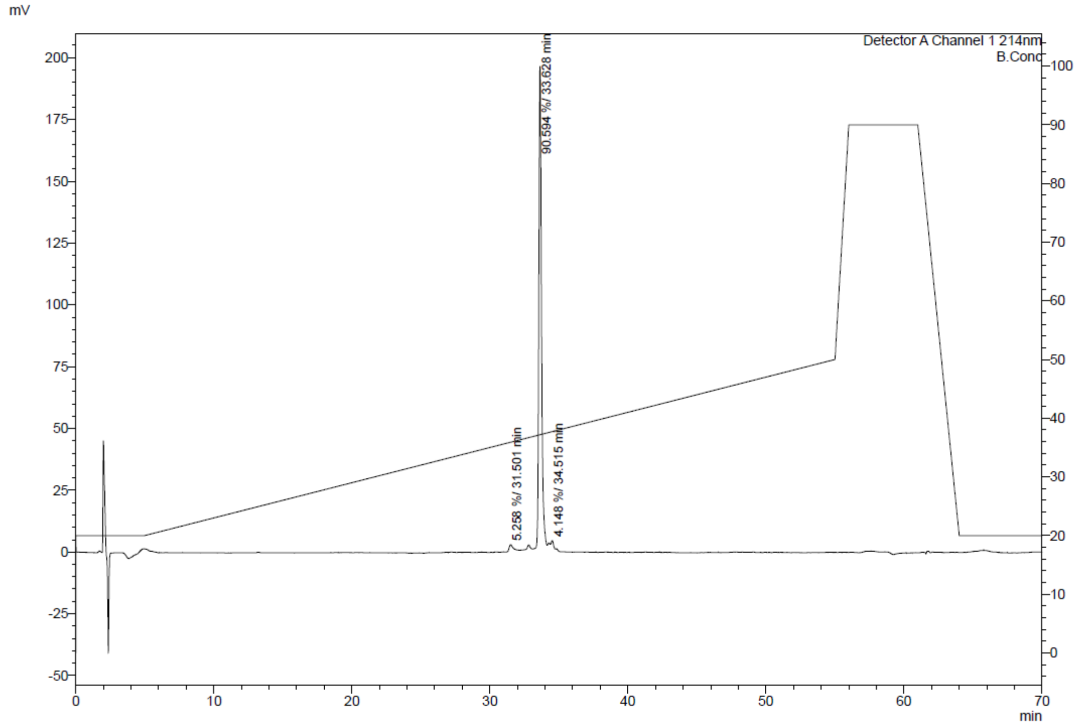

*SI Figure 9: 50 min gradient 20-50% B HPLC for peptide **8a**.*

Peptide 9a

32.62 min 96.25% (50 min gradient, 20-50% B).

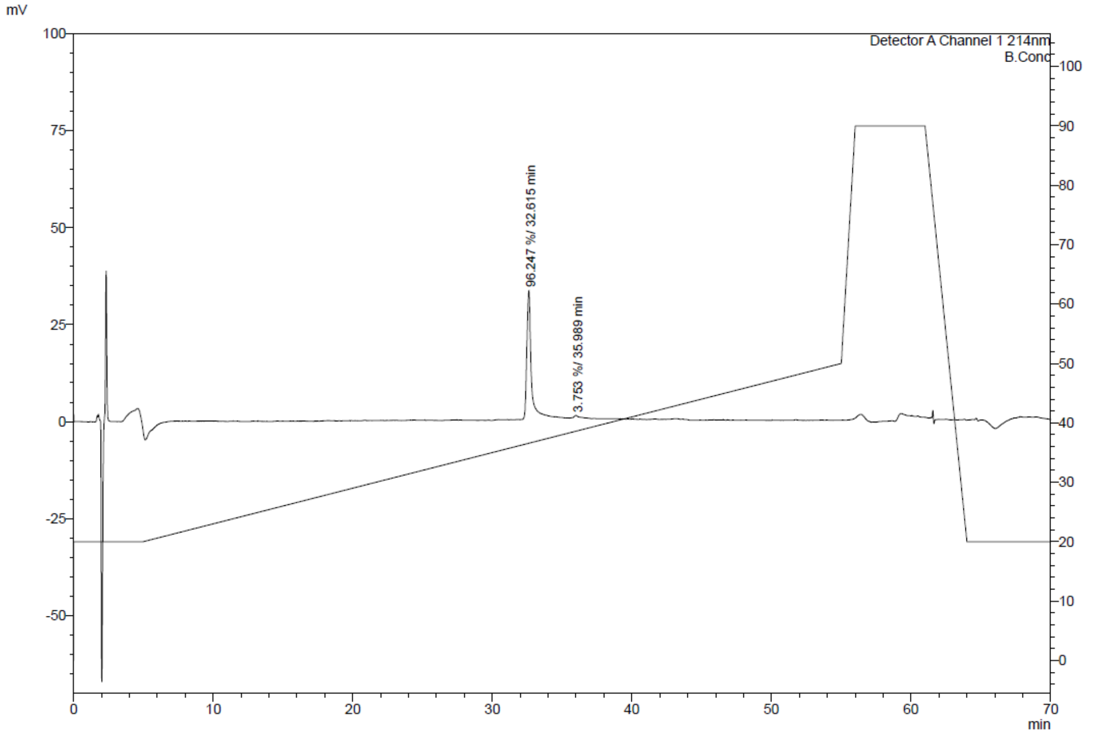

*SI Figure 10: 50 min gradient 20-50% B HPLC for peptide **9a**.*

Peptide 10a
35.17 min 100% (50 min gradient, 20-50% B).

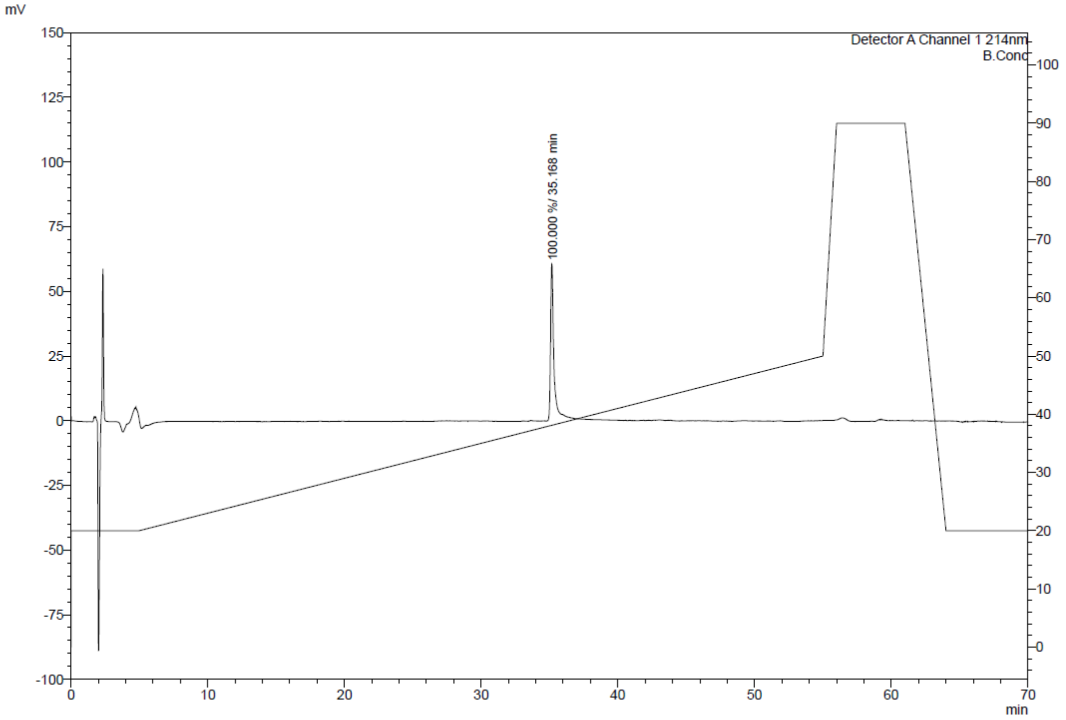

*SI Figure 11: 50 min gradient 20-50% B HPLC for peptide **10a**.*

Peptide 11a
18.14 min 97.02% (50 min gradient, 30-60% B).

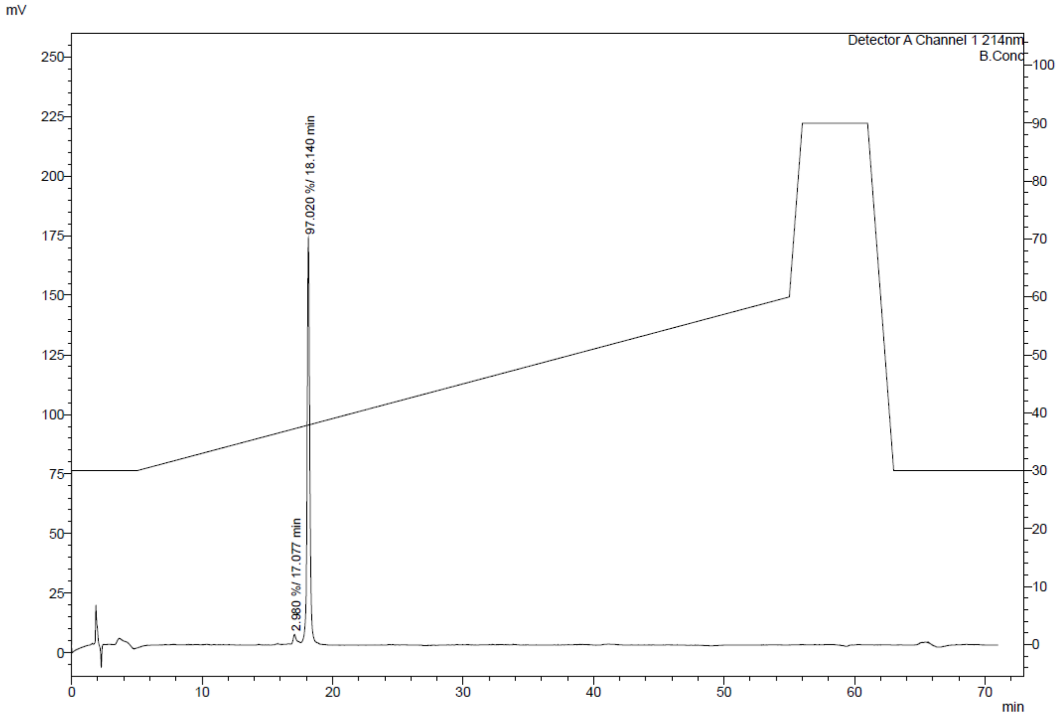

*SI Figure 12: 50 min gradient 30-60% B HPLC for peptide **11a**.*

Peptide 1b

13.53 min 95.68% (50 min gradient, 30-60% B).

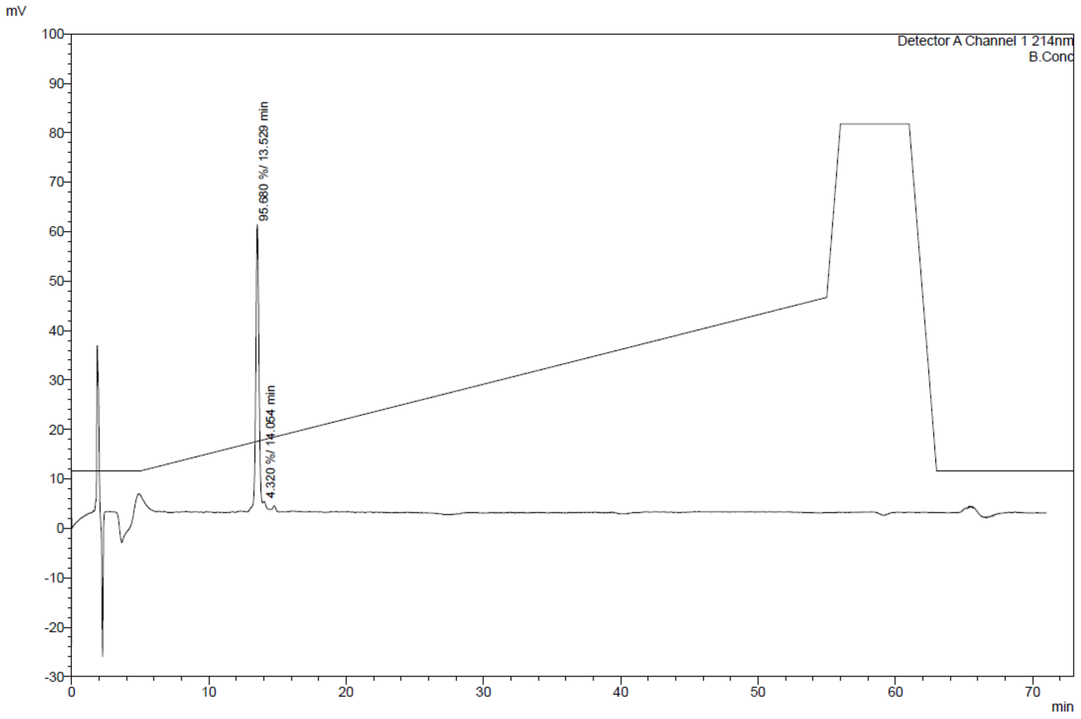

*SI Figure 13: 50 min gradient 30-60% B HPLC for peptide **1b**.*

Peptide 2b

26.89 min 98.43% (50 min gradient, 20-50% B).

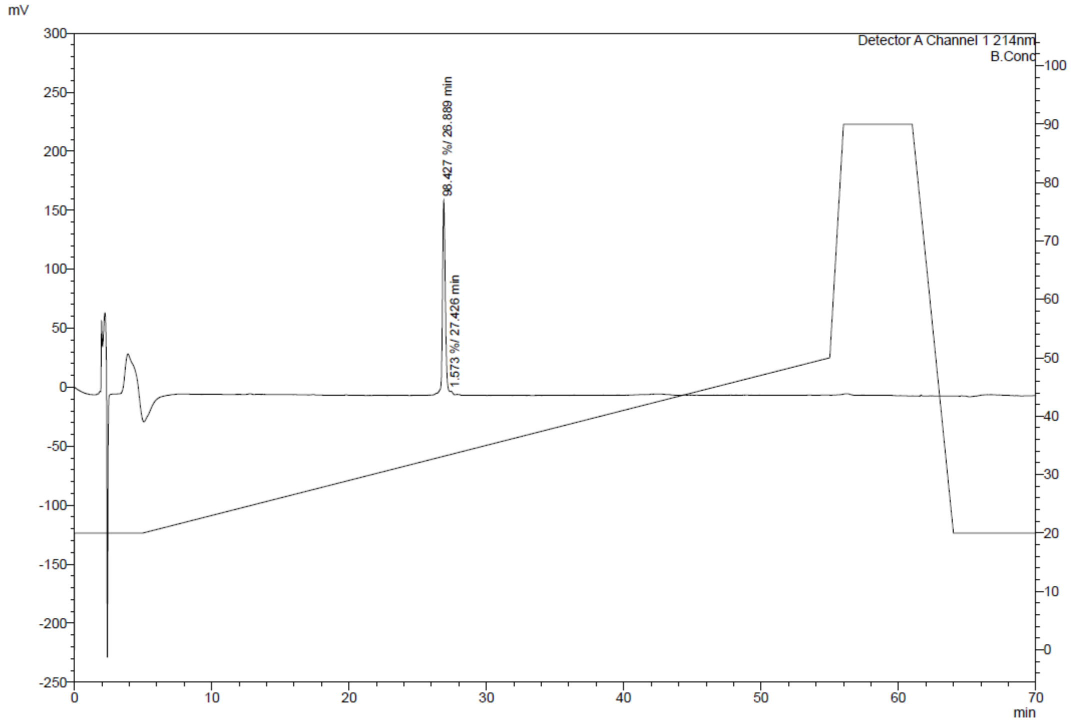

*SI Figure 14: 50 min gradient 20-50% B HPLC for peptide **2b**.*

Peptide 3b
24.39 min 89.50% (50 min gradient, 20-50% B).

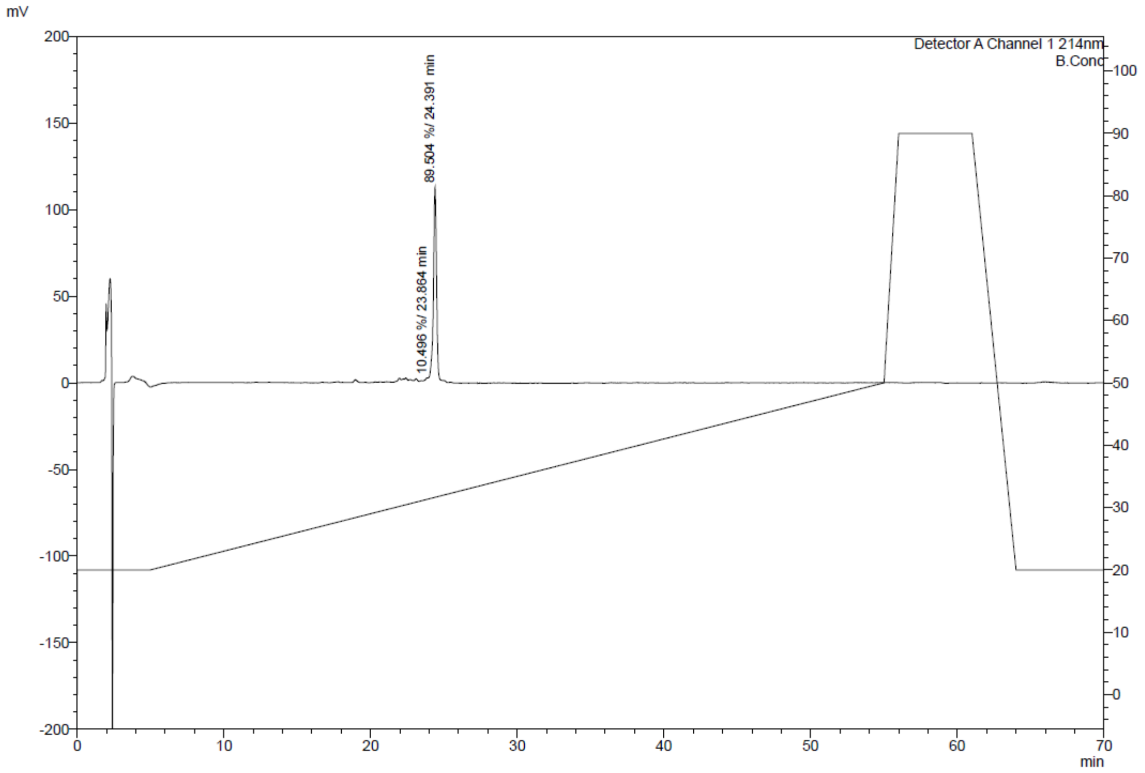

*SI Figure 15: 50 min gradient 20-50% B HPLC for peptide **3b**.*

Peptide 4b
23.26 min 96.38% (50 min gradient, 20-50% B).

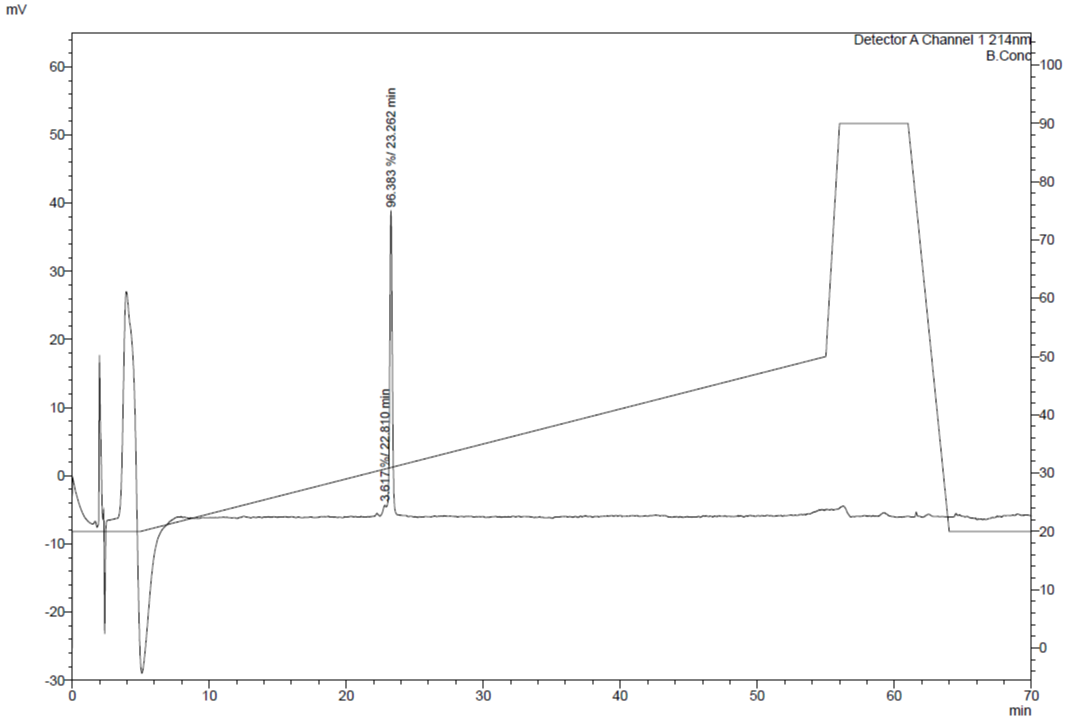

*SI Figure 16: 50 min gradient 20-50% B HPLC for peptide **4b**.*

Peptide 5b

27.75 min 96.79% (50 min gradient, 20-50% B).

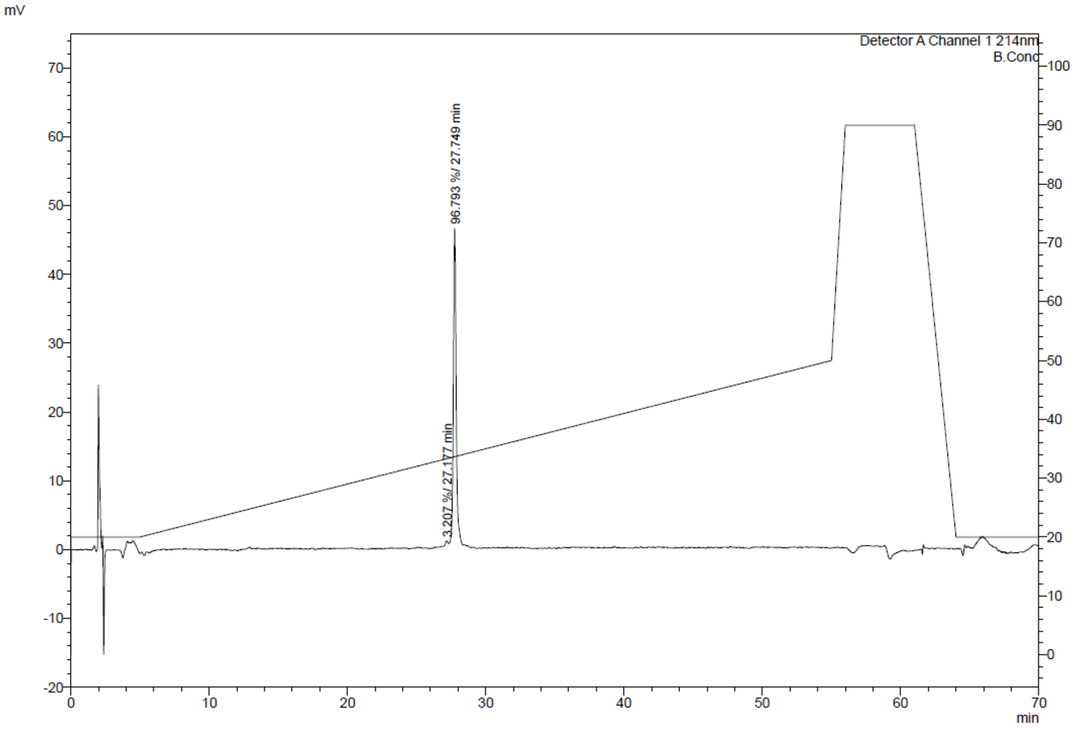

*SI Figure 17: 50 min gradient 20-50% B HPLC for peptide **5b**.*

Peptide 6b

25.73 min 93.72% (50 min gradient, 20-50% B).

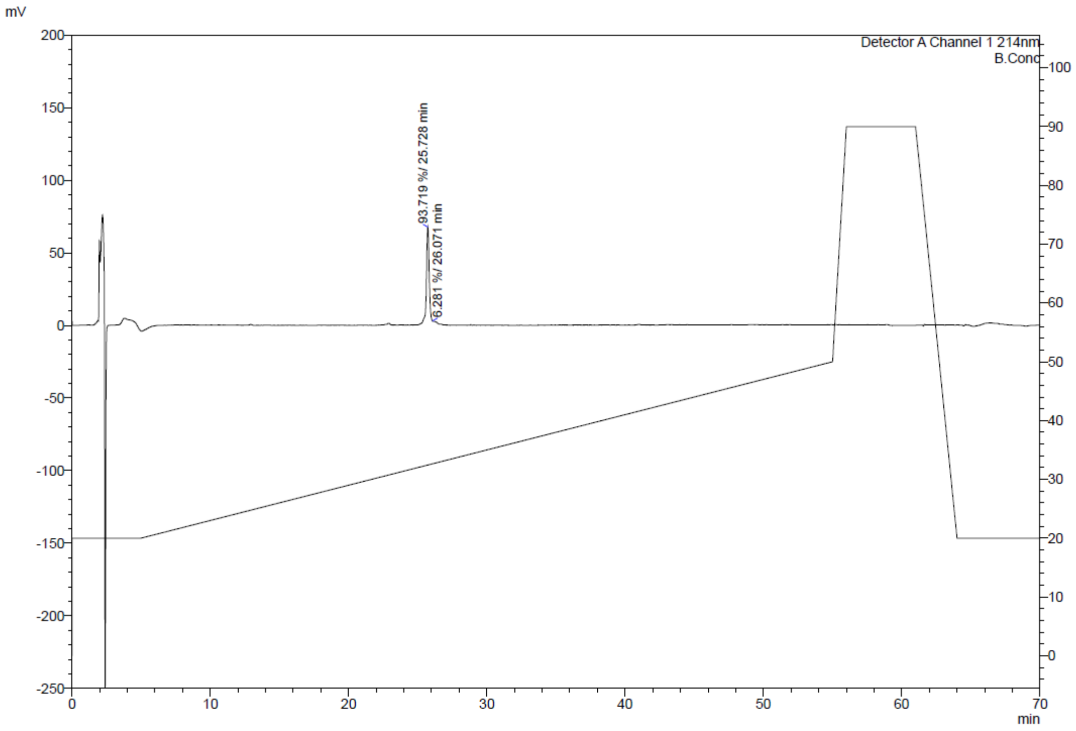

*SI Figure 18: 50 min gradient 20-50% B HPLC for peptide **6b**.*

Peptide 7b
26.36 min 95.04% (50 min gradient, 20-50% B).

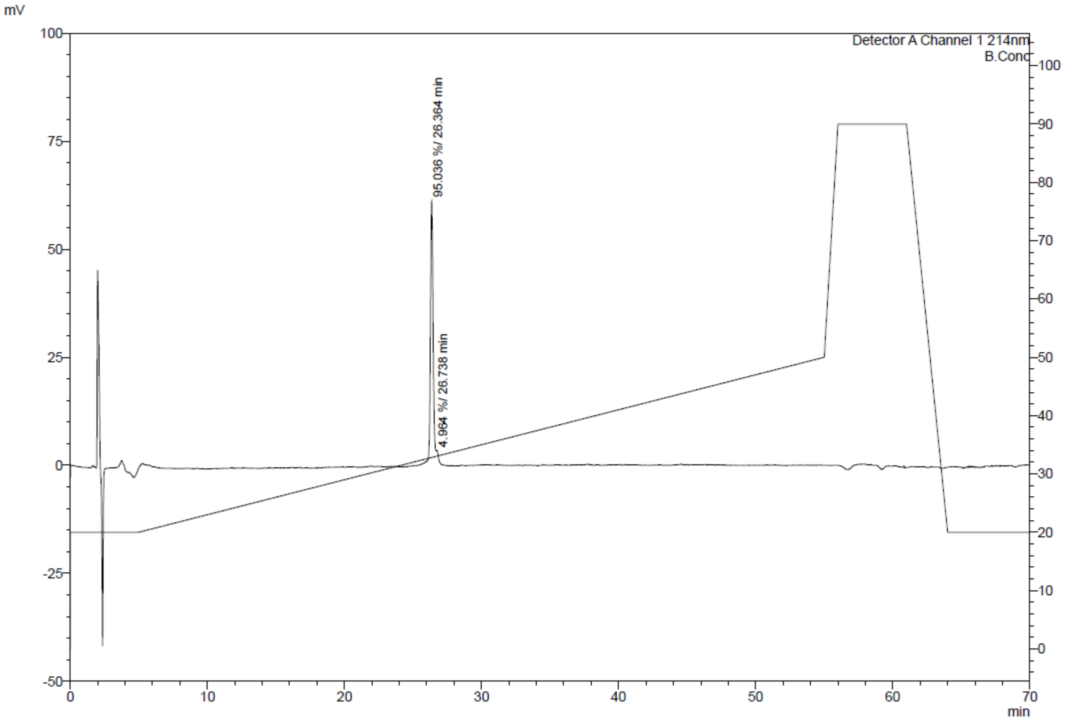

*SI Figure 19: 50 min gradient 20-50% B HPLC for peptide **7b**.*

Peptide 8b
27.37 min 100% (50 min gradient, 20-50% B).

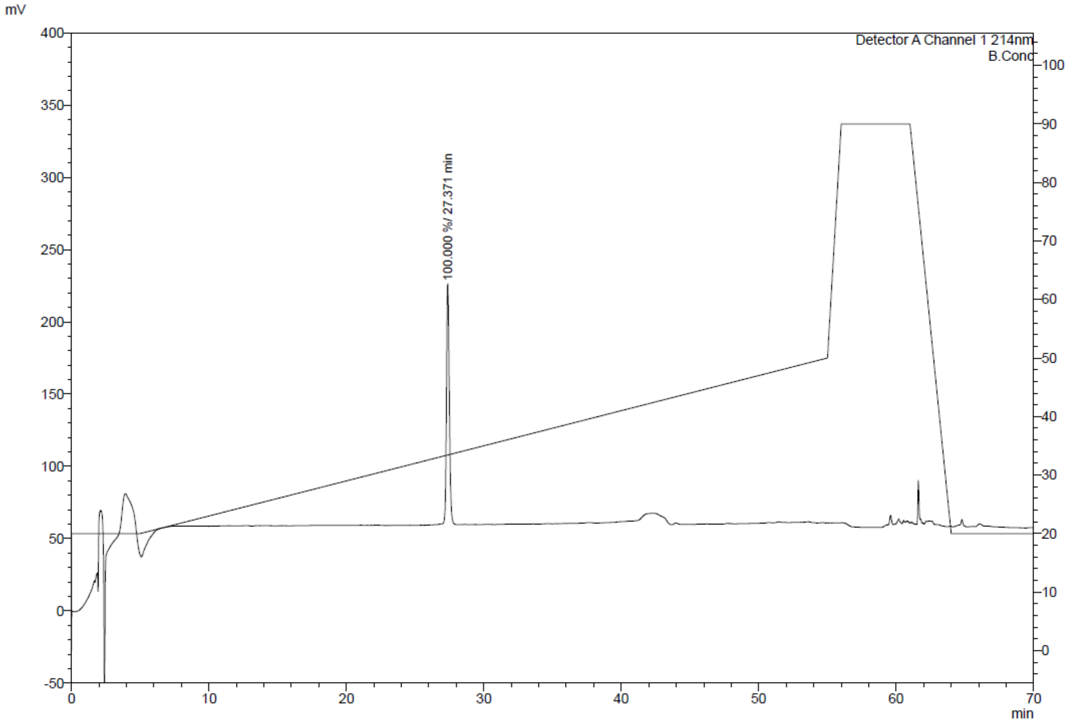

*SI Figure 20: 50 min gradient 20-50% B HPLC for peptide **8b**.*

Peptide 9b
26.24 min 92.93% (50 min gradient, 20-50% B).

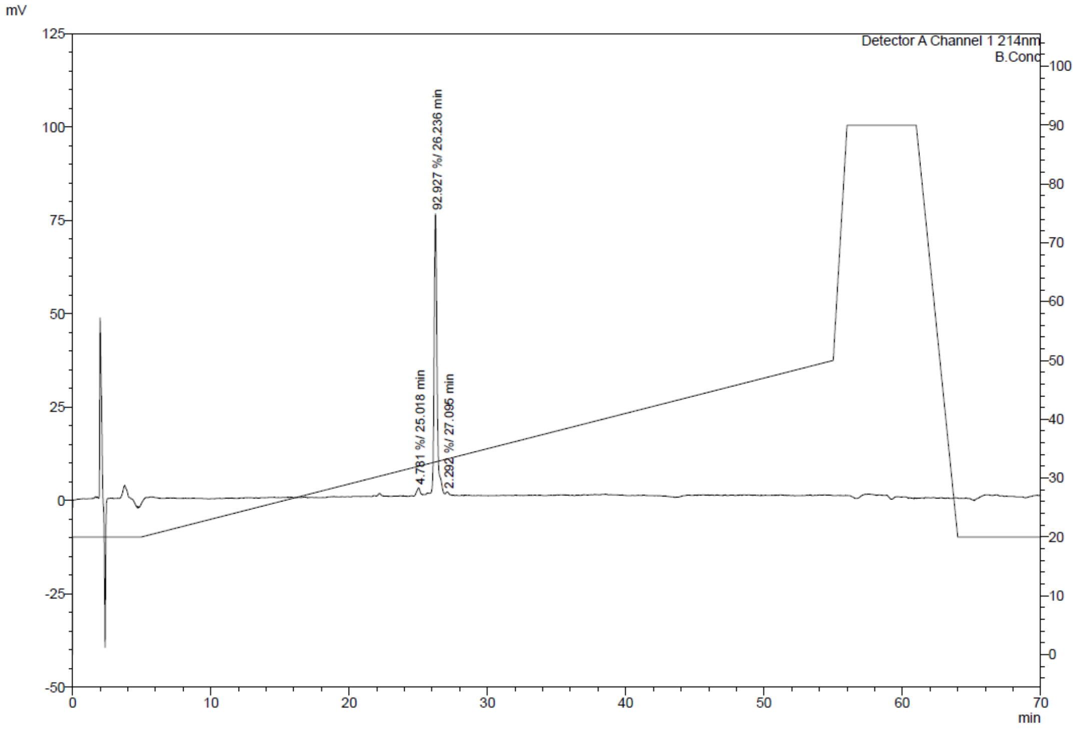

*SI Figure 21: 50 min gradient 20-50% B HPLC for peptide **9b**.*

Peptide 10b
28.97 min 100% (50 min gradient, 30-60% B).

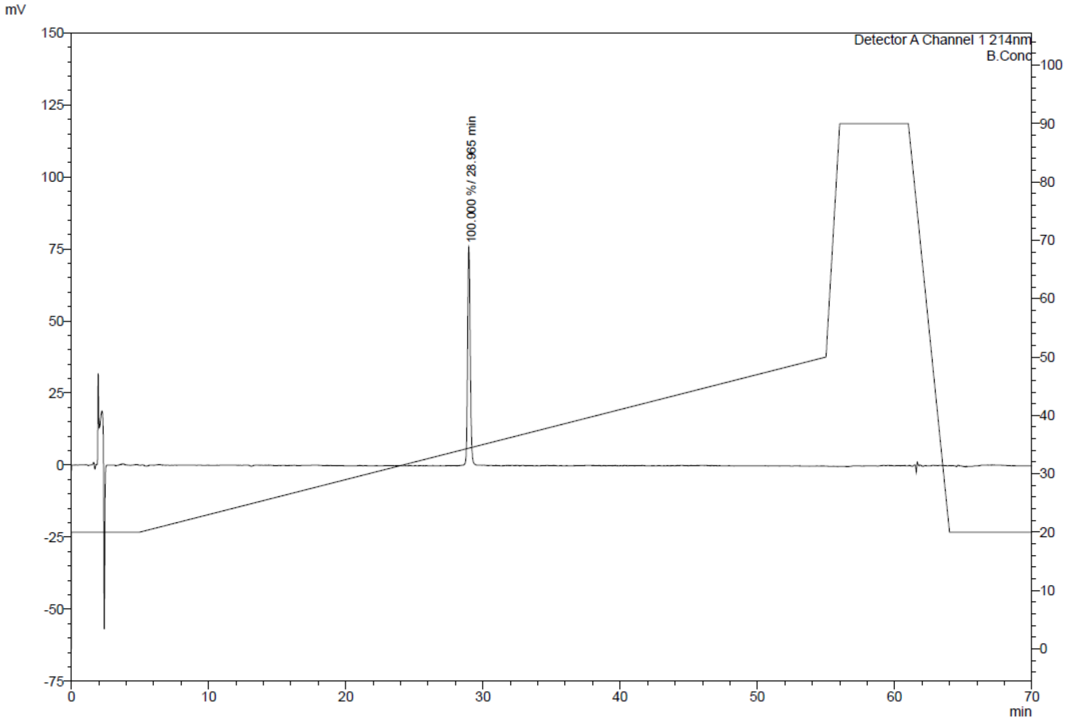

*SI Figure 22: 50 min gradient 30-60% B HPLC for peptide **10b**.*

Peptide 11b

28.96 min 100% (50 min gradient, 20-50% B).

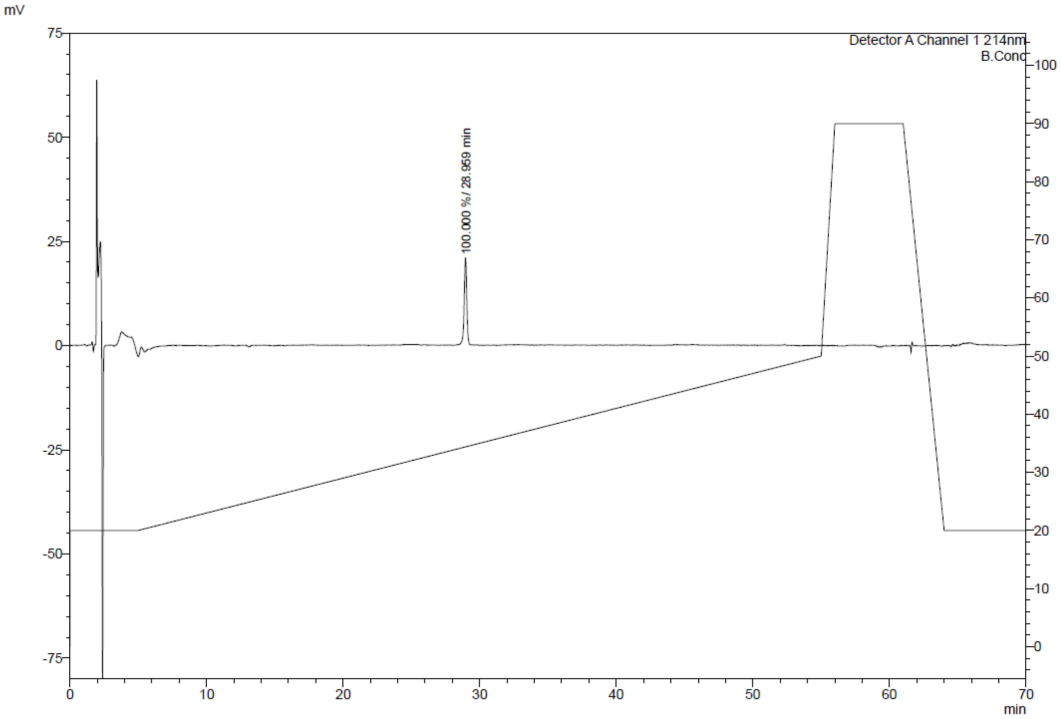

*SI Figure 23: 50 min gradient 20-50% B HPLC for peptide **11b**.*

Fluorescence polarization fits

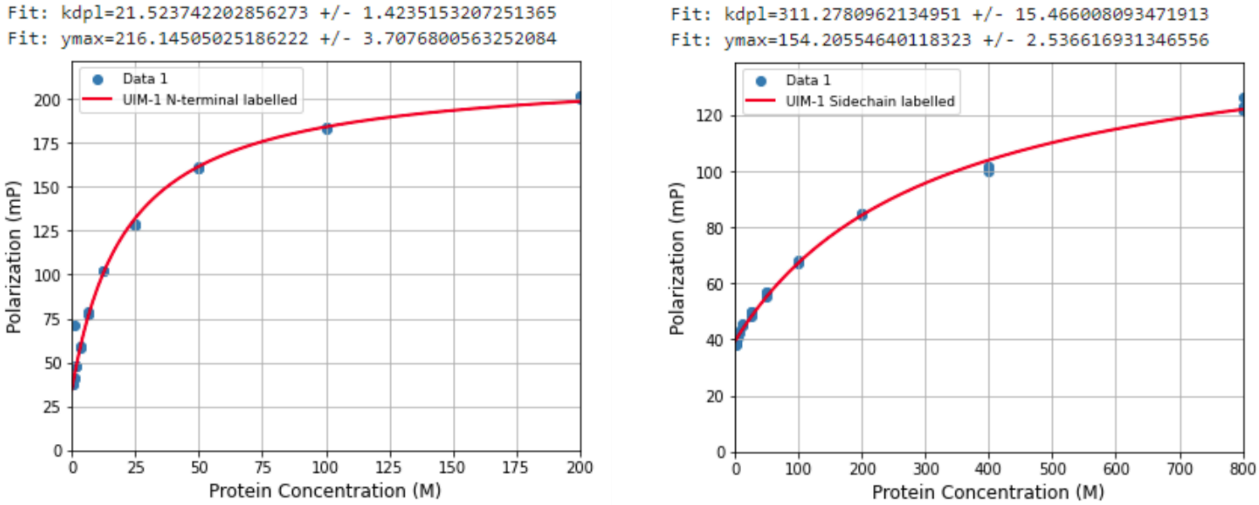

Figure S24: FP assay fits for peptides **1a** (left) and **1c**

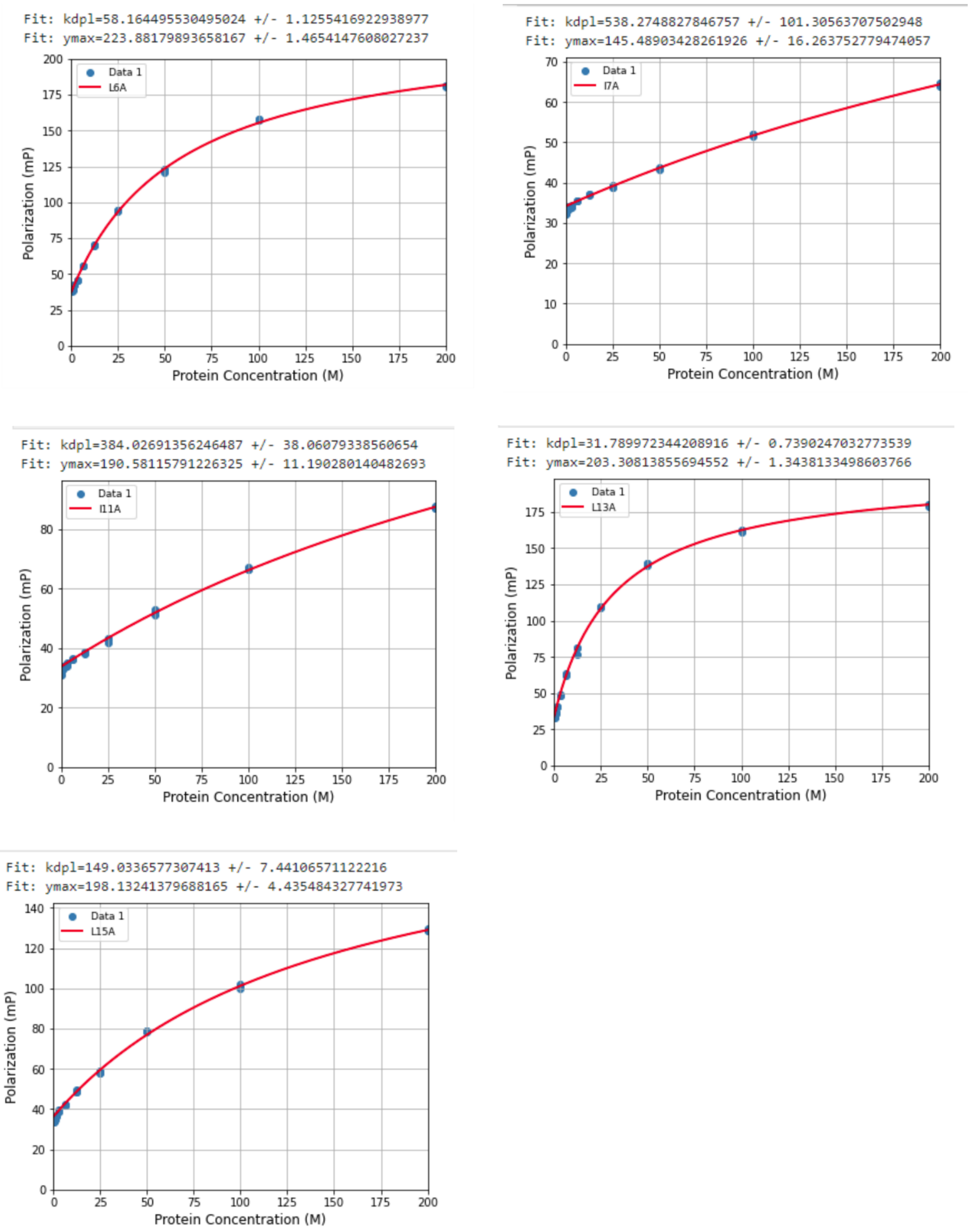

Figure S25: FP assay fits for peptides **2a**, **3a**, **4a**, **5a** and **6a**.

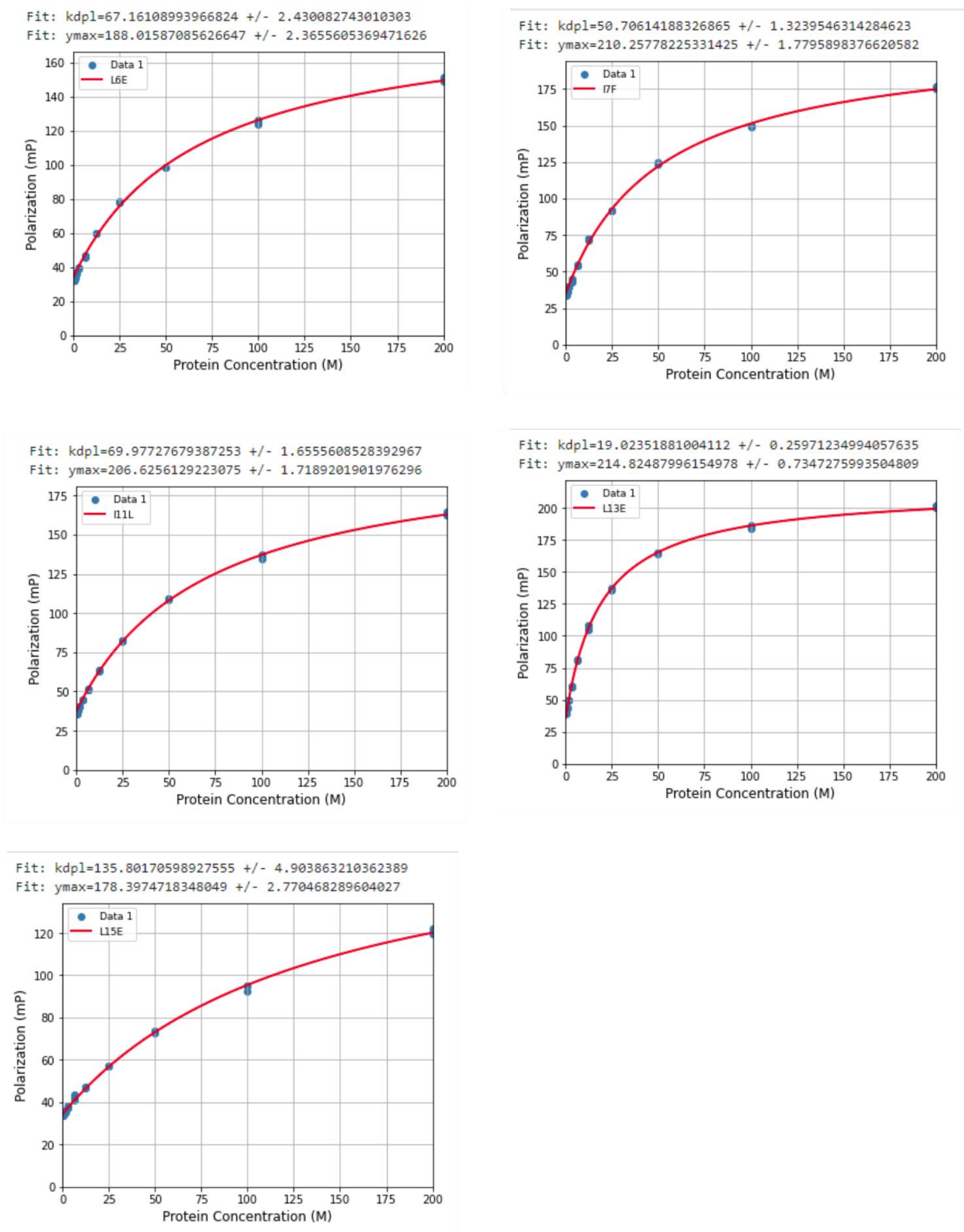

Figure S26: FP assay fits for peptides **7a**, **8a**, **9a**, **10a** and **11a**.

SI References

(1) Zubarev, R. A.; Håkansson, P.; Sundqvist, B. Accuracy Requirements for Peptide Characterization by Monoisotopic Molecular Mass Measurements. *Anal. Chem.* **1996**, *68* (22), 4060–4063. https://doi.org/10.1021/ac9604651.

(2) Cody, R. B. Why Are We Still Reporting Mass Accuracy in Parts per Million (Ppm)? *J. Am. Soc. Mass Spectrom.* **2020**, *31* (4), 1004–1005. https://doi.org/10.1021/jasms.9b00150.

(3) Dou, H.; Buetow, L.; Hock, A.; Sibbet, G. J.; Vousden, K. H.; Huang, D. T. Structural Basis for Autoinhibition and Phosphorylation-Dependent Activation of c-Cbl. *Nat. Struct. Mol. Biol.* **2012**, *19* (2), 184–192. https://doi.org/10.1038/nsmb.2231.

(4) Moerke, N. J. Fluorescence Polarization (FP) Assays for Monitoring Peptide-Protein or Nucleic Acid-Protein Binding. *Curr. Protoc. Chem. Biol.* **2009**, *1* (1), 1–15. https://doi.org/10.1002/9780470559277.ch090102.

(5) Mirdita, M.; Schütze, K.; Moriwaki, Y.; Heo, L.; Ovchinnikov, S.; Steinegger, M. ColabFold: Making Protein Folding Accessible to All. *Nat. Methods* **2022**, *19* (6), 679–682. https://doi.org/10.1038/s41592-022-01488-1.

(6) Lambrughi, M.; Maiani, E.; Fas, B. A.; Shaw, G. S.; Kragelund, B. B.; Lindorff-Larsen, K.; Teilum, K.; Invernizzi, G.; Papaleo, E. Ubiquitin Interacting Motifs: Duality Between Structured and Disordered Motifs. *Front. Mol. Biosci.* **2021**, *8* (June), 1–14. https://doi.org/10.3389/fmolb.2021.676235.
